# Copy-scAT: Deconvoluting single-cell chromatin accessibility of genetic subclones in cancer

**DOI:** 10.1126/sciadv.abg6045

**Published:** 2021-10-13

**Authors:** Ana Nikolic, Divya Singhal, Katrina Ellestad, Michael Johnston, Yaoqing Shen, Aaron Gillmor, Sorana Morrissy, J. Gregory Cairncross, Steven Jones, Mathieu Lupien, Jennifer A. Chan, Paola Neri, Nizar Bahlis, Marco Gallo

**Affiliations:** 1 Arnie Charbonneau Cancer Institute, Cumming School of Medicine, University of Calgary, Calgary, AB, Canada.; 2 Alberta Children’s Hospital Research Institute, Cumming School of Medicine, University of Calgary, Calgary, AB, Canada.; 3 Department of Biochemistry and Molecular Biology, Cumming School of Medicine, University of Calgary, Calgary, AB, Canada.; 4 Canada’s Michael Smith Genome Sciences Centre, British Columbia Cancer Agency, Vancouver, BC, Canada.; 5 Department of Medical Genetics, University of British Columbia, Vancouver, BC, Canada.; 6 Department of Oncology, Cumming School of Medicine, University of Calgary, Calgary, AB, Canada.; 7 Princess Margaret Cancer Centre, University Health Network, Toronto, ON, Canada.; 8 Department of Medical Biophysics, University of Toronto, Toronto, ON, Canada.; 9 Ontario Institute for Cancer Research, Toronto, ON, Canada.

## Abstract

Single-cell epigenomic assays have tremendous potential to illuminate mechanisms of transcriptional control in functionally diverse cancer cell populations. However, application of these techniques to clinical tumor specimens has been hampered by the current inability to distinguish malignant from nonmalignant cells, which potently confounds data analysis and interpretation. Here, we describe Copy-scAT, an R package that uses single-cell epigenomic data to infer copy number variants (CNVs) that define cancer cells. Copy-scAT enables studies of subclonal chromatin dynamics in complex tumors like glioblastoma. By deploying Copy-scAT, we uncovered potent influences of genetics on chromatin accessibility profiles in individual subclones. Consequently, some genetic subclones were predisposed to acquire stem-like or more differentiated molecular phenotypes, reminiscent of developmental paradigms. Copy-scAT is ideal for studies of the relationships between genetics and epigenetics in malignancies with high levels of intratumoral heterogeneity and to investigate how cancer cells interface with their microenvironment.

## Teaser

Inferring structural variants in cancer single-cell chromatin accessibility data links genetics to chromatin organization.

## INTRODUCTION

Single-cell genomic technologies have made enormous contributions to the deconvolution of complex cellular systems, including cancer (*1*). Single-cell RNA sequencing (scRNA-seq), particularly, has been widely used to understand the implications of intratumoral transcriptional heterogeneity for tumor growth, response to therapy, and patient prognosis (*2*–*6*). This field has greatly benefited from an emerging ecosystem of computational tools that have enabled complex analyses of scRNA data. Because copy number variants (CNVs) mostly accrue in malignant cells and are rare in nonmalignant tissues, computational platforms that use scRNA data to call CNVs have resulted in improved understanding of the behavior of genetic subclones in tumors (*7*–*9*).

On the other hand, the application of single-cell epigenomic techniques, including the assay for transposase accessible chromatin (scATAC) (*10*, *11*), to study cancer has been slowed by computational bottlenecks. For instance, unlike scRNA-seq, now, no dedicated tool exists to call CNVs using scATAC data. This technical gap has slowed the use of scATAC to study clinical tumor specimens, which often are surgical resections that include both malignant and nonmalignant cells. Inability to deconvolute these cell populations after the generation of scATAC libraries would confound downstream analyses and interpretation of this data type.

Here, we describe Copy-scAT [copy number inference using scATAC sequencing (scATAC-seq) data], a new computational tool that uses scATAC datasets to call CNVs at the single-cell level. Using scATAC datasets from adult glioblastoma (aGBM), pediatric GBM (pGBM), and multiple myeloma (MM), we demonstrate the effectiveness of Copy-scAT in calling (i) focal amplifications and (ii) chromosome arm-level gains and losses. At the most basic level, Copy-scAT can therefore discriminate between malignant and nonmalignant cells in scATAC datasets based on the presence or absence, respectively, of CNVs. This distinction is fundamental to ensure that downstream analyses include only the appropriate tumor or microenvironment cell populations. Furthermore, application of Copy-scAT allows the relationship between genetic and epigenetic differences to be investigated within individual subclones. In this regard, we show that cells that share a given CNV tend to cluster together in scATAC experiments, suggesting that genetics may impart information that results in the emergence of specific epigenetic profiles. We illustrate this principle by providing examples of coexisting genetic subclones with stem-like or more differentiated molecular profiles in GBM.

## RESULTS

### Design and implementation of copy-scAT

**Fig. 1.**
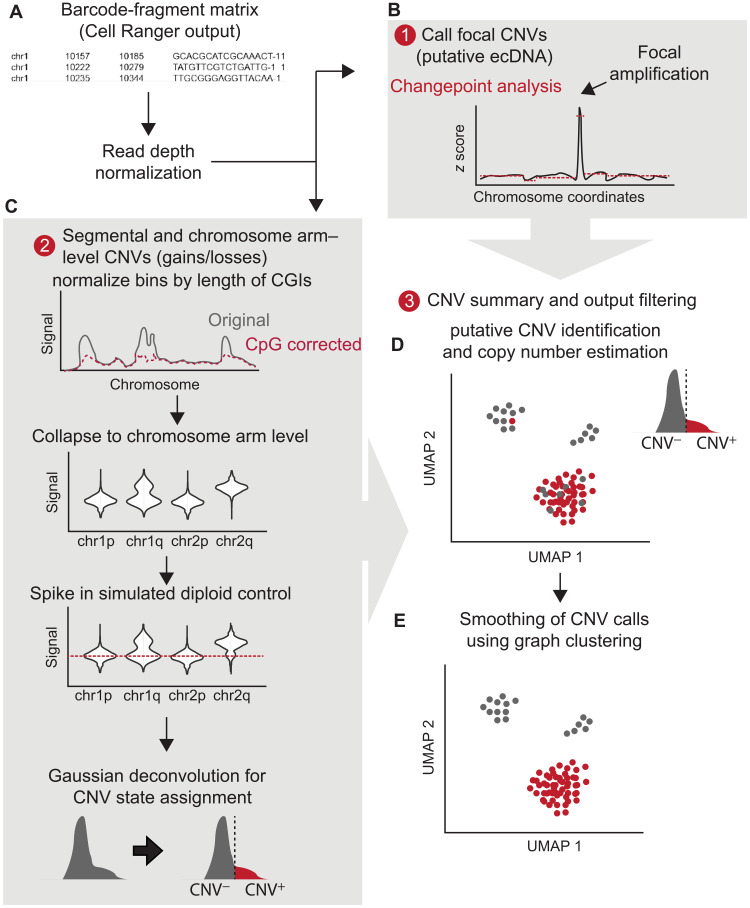
Copy-scAT workflow. (**A**) Copy-scAT accepts barcode-fragment matrices generated by Cell Ranger (10x Genomics) as input.(**B**) Large peaks in normalized coverage matrices can be used to infer focal CNVs. ecDNA, extrachromosomal DNA. (**C**) Normalized matrices can be used to infer segmental and chromosome arm-level CNVs. (**D**) Example of chromosome arm-level CNV (chromosome 10p loss) called by Copy-scAT. (**E**) Consensus clustering is used to finalize cell assignment.

We designed Copy-scAT, an R package that uses scATAC-seq information to infer copy number alterations. Copy-scAT uses fragment files generated by cellranger-atac (10x Genomics) as input to generate chromatin accessibility pileups, keeping only barcodes with a minimum number of fragments (defaulting to 5000 fragments). It then generates a pileup of total coverage (number of reads × read lengths) over bins of determined length (1 Mbp as default) (Fig. 1A). Binned read counts then undergo linear normalization over the total signal in each cell to account for differences in read depth, and chromosomal bins that consist predominantly of zeros (at least 80% zero values) are discarded from further analysis. All parameters, including reference genome, bin size, and minimum length cutoff, are user-customizable. Copy-scAT then implements different algorithms to detect focal amplifications and larger-scale copy number variation.

To call focal amplifications (Fig. 1B), Copy-scAT generates a linear scaled profile of density over normalized 1-Mbp bins along each chromosome on a single-cell basis, centering on the median and scaling using the range. Copy-scAT then uses changepoint analysis using mean and variance (see Materials and Methods) (*12*) to identify segments of abnormally high signal (default threshold uses *z* score > 5) along each chromosome in each single cell. Optimization of settings shows that this particular threshold filters out most spurious calls, and the use of mean variance for determination of changepoint improves the detection of consensus regions because of lesser effects of low-coverage and inaccessible regions (fig. S1). These calls are then pooled together to generate consensus regions of amplification, a strategy that could enable identification of putative double minutes and extrachromosomal DNA amplifications. Each cell is then scored as positive or negative for each amplified genomic region.

For larger copy number alterations, Copy-scAT pools the bins further at the chromosome arm level using a trimmed mean (keeping all bins between the 50th and 75th percentiles as default) while normalizing the data on the basis of length of CpG islands contained in each bin (Fig. 1C). Data are then scaled for each chromosome arm compared to a pseudodiploid control (expected signal distribution for a diploid genotype) that is modeled for each sample, and cluster assignments are generated using Gaussian decomposition. Cluster assignments are then normalized to get an estimate of copy number for each cell (Fig. 1D). These assignments can be optionally smoothed using alternative clustering information (Louvain clustering by default, although other clustering methods such as *k*-means may also be used) to generate consensus genotypes for each cluster of cells, improving accuracy for cells with sparse coverage (Fig. 1E). For full details regarding the execution of Copy-scAT, see Materials and Methods. A step-by-step tutorial for Copy-scAT is available on GitHub (see Materials and Methods).

### Copy-scAT effectively calls CNVs in diverse malignancies

**Fig. 2.**
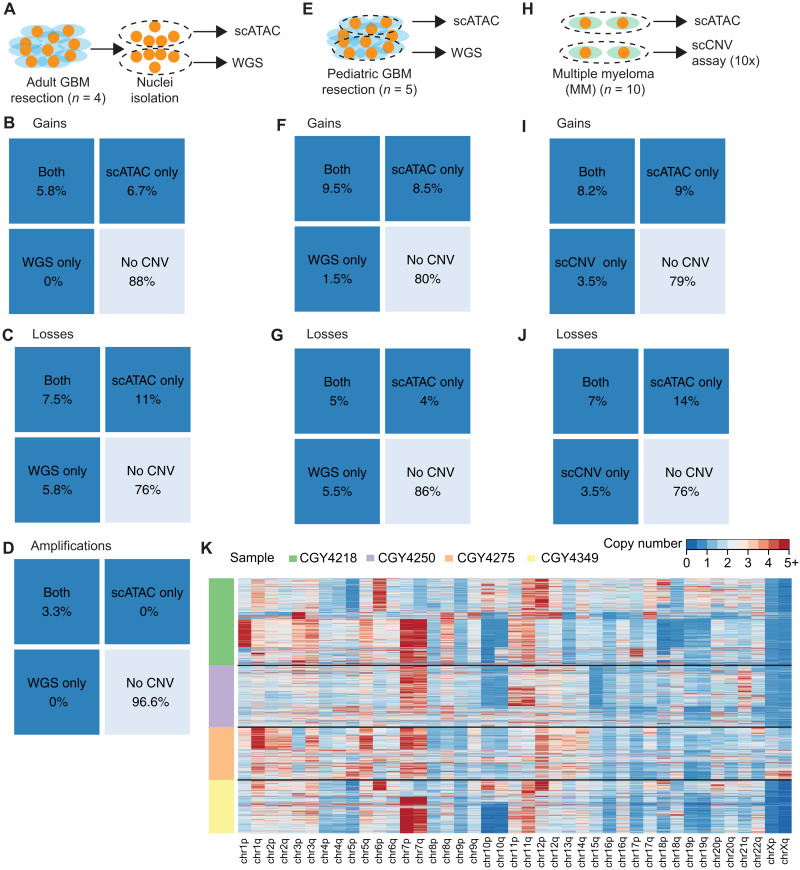
Benchmarking of Copy-scAT with three methods involving clinical samples from three distinct malignancies. (**A**) Banked cryopreseved aGBM samples were used for both scATAC and WGS. Nuclei were isolated from the samples, mixed, and used for both scATAC and WGS. (**B** to **D**) Percent of chromosome arm-level gains, chromosome arm-level losses, and focal amplifications detected in aGBM samples identified using both methods versus CNVs detected by scATAC or WGS alone. (**E**) Banked frozen pGBM samples were used for both scATAC and WGS. (**F** and **G**) Number of chromosome arm-level gains and losses detected in pGBM samples identified using both methods versus total numbers of gains detected by scATAC or WGS. (**H**) Fresh MM samples were used for both scATAC and 10x scCNV DNA analysis. (**I** and **J**) Number of chromosome arm-level gains and losses detected by both methods versus total numbers of gains detected by scATAC or 10x scCNV analysis. (**K**) Overview of raw transformed *z*-score profiles for four aGBM samples.

We have tested the ability of Copy-scAT to use scATAC data to call CNVs with three different approaches and with different tumor types. First, we benchmarked Copy-scAT against CNV calls made with whole-genome sequencing (WGS) data for aGBM surgical resections (*n* = 4 samples, 4878 cells). This approach consisted in isolating nuclei from cryopreserved aGBM samples, mixing nuclei in suspension, and then using these nuclei for either scATAC or WGS library construction (Fig. 2A). This was meant to ensure similar representation of genetic subclones, which are usually regionally contiguous in this solid tumor, in both scATAC and WGS libraries. Confusion matrices were generated to compare chromosome arm-level gains, chromosome arm-level losses, and focal amplifications inferred by Copy-scAT, WGS, or both (Fig. 2, B to D). Percentages were determined as number of chromosome arms with an alteration over all chrosomome arms in all samples (see Materials and Methods). Second, we benchmarked Copy-scAT against CNV calls made using pGBM surgical resections (*n* = 5 longitudinally collected samples from three different patients, 10,574 cells). In this case, scATAC and WGS libraries were generated from separate geographical regions of the same tumor (Fig. 2, E to G). Third, we benchmarked Copy-scAT against CNV calls made with the single-cell CNV (scCNV) assay (10x Genomics) using MM clinical samples (*n* = 10 samples, 44,161 cells) (Fig. 2, H to J, and fig. S4). Overall, we observed that Copy-scAT correctly inferred all or most of the CNVs that were called with WGS (Fig. 2, A to G, and figs. S2 and S3) or scCNV data (Fig. 2, H to J, and fig. S4). In total, we profiled 59,613 cells from 19 malignancies from 17 patients and were able to infer CNV status for a total of 52,123 cells (table S1). On average, we were able to call CNVs for 86.49% of cells in each sample (range, 81.77 to 93.35%) (table S1).

For chromosome arm-level CNV gains, precision was moderate, averaging 0.55 over all samples (range, from 0.47 in pGBM to 0.57 in myeloma), and recall was very good (average, 0.84; range, 0.77 from MM to 1.0 in aGBM) (table S2). Accuracy was approximately 0.84 (range, 0.82 to 0.87), assuming WGS as gold standard, suggesting that we were able to retrieve most of the copy number gains detected by WGS or scCNV analysis. For chromosome arm-level losses, precision was again moderate (0.53; range, 0.43 to 0.72 for different sample types), recall was 0.84 (range, 0.66 to 0.75 for different sample types), and accuracy was 0.79 (range, 0.72 to 1.0 for different sample types) (table S2). The sensitivity and specificity of focal amplifications were excellent, with perfect concordance between WGS and scATAC; however, no amplifications were detected in the profiled pGBM and MM samples, so the number of alterations detected overall was much smaller (table S2).

The variation observed may reflect technical differences between the strategies used for benchmarking. The recall and accuracy were slightly lower in the MM samples, but this may, in part, be due to the relatively lower number of reads per cell for these samples, leading to greater noise, and the relative paucity of normal cells compared to brain tumors (fig. S5). As expected, the calls of Copy-scAT for aGBM were the most accurate, likely because scATAC and WGS datasets were generated by relatively homogeneous starting material, as described above. Because of its design, it is also possible that Copy-scAT is more sensitive at inferring CNVs that occur in relatively rare subclones compared to WGS, potentially explaining (in addition to true false positives) why the precision metrics are lower than recall and accuracy for our tool, as the number of CNVs inferred by Copy-scAT is often higher than the number of inferences made with WGS.

Copy number calls made with Copy-scAT can be used to visualize genetic heterogeneity in clinical samples. As illustrative examples, raw heatmaps of imputed copy number for aGBM samples show evidence of interpatient and intratumoral heterogeneity (Fig. 2K). Some of the most common CNVs inferred by Copy-scAT in these samples, particularly chromosome 7 gains and chromosome 10 losses, are hallmarks of aGBM. Overall, our results showcase the ability of Copy-scAT to use scATAC data to infer CNVs.

### scATAC data can be used to distinguish malignant from nonmalignant cells

**Fig. 3.**
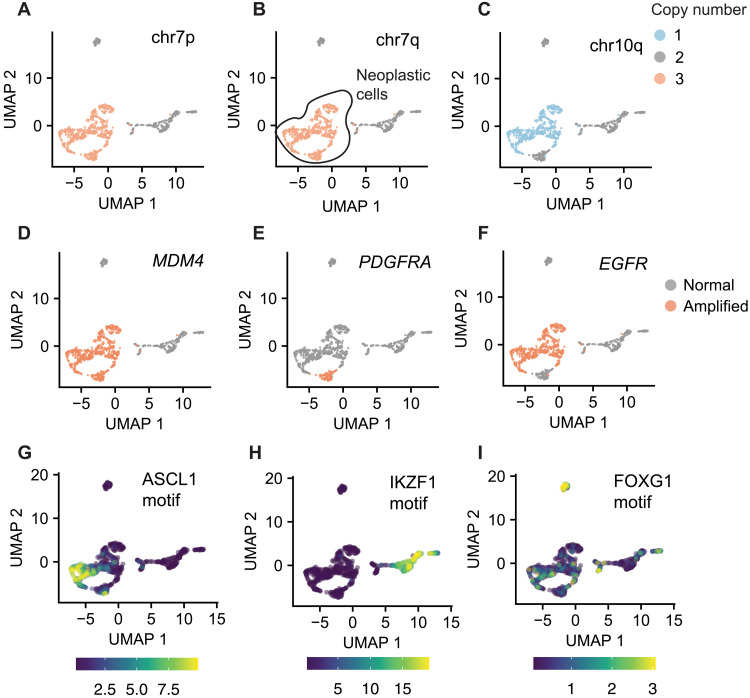
Detection of CNVs and identification of neoplastic clones with Copy-scAT. (**A**) Chromosome 7p gain in an aGBM sample (CGY4349). (**B**) Chromosome 7q gain in an aGBM sample. (**C**) Chromosome 10p loss in an aGBM sample. (**D**) *MDM4* amplification in an aGBM sample (CGY4349). Amplified cells, orange; nonamplified cells, gray. (**E**) *PDGFRA* amplification in an aGBM sample (CGY4349). Amplified cells, orange; nonamplified cells, gray. (**F**) *EGFR* amplification in an aGBM sample (CGY4349). Amplified cells, orange; nonamplified cells, gray. (**G**) ChromVAR activity score for the ASCL1 motif. (**H**) ChromVAR activity score for the IKZF1 motif. (**I**) ChromVAR activity score for the FOXG1 motif.

Tumor cells often harbor CNVs, and we reasoned that the use of Copy-scAT should enable the use of scATAC data to infer CNVs and therefore distinguish between malignant and nonmalignant cells. To test this hypothesis, we overlayed CNVs called by Copy-scAT onto scATAC datasets displayed in uniform manifold approximation and projection (UMAP) plots. This exercise led to the identification of cells that were clearly positive for multiple CNVs typical of GBM and others that appeared to have a normal genome (Fig. 3, A to C). Globally, both copy number alterations and amplifications showed strong concordance within UMAP-defined clusters, even before smoothing, in keeping with a strong delineation of subclones, with minimal changes after smoothing (figs. S6 and S7). As an illustrative example, we found that the aGBM sample CGY4349 was composed of discrete cell populations with chromosome 7 gain (Fig. 3, A and B), chromosome 10p deletion (Fig. 3C), and along with focal amplifications at the *MDM4* (Fig. 3D), *PDGFRA* (Fig. 3E), and *EGFR* (Fig. 3F) loci. Copy-scAT results suggest specific lineage relationships between subclones. For instance, chromosome 7 amplifications are clonal in this sample (Fig. 3, A and B), whereas the chromosome 10 deletion is subclonal (Fig. 3C). In addition, our computational tool predicts that *PDGFRA* (Fig. 3E) and *EGFR* (Fig. 3F) focal amplifications are mutually exclusive, a phenomenon that has been reported for aGBM (*13*).

Together, these results illustrate one specific population of cells (circled in black in Fig. 3B) that harbors several CNVs and are therefore putative cancer cells. At the same time, we also identified cells (labeled in gray in Fig. 3B) that did not appear to have any CNVs and are therefore likely to be nonneoplastic cells from the tumor microenvironment. Equivalent results were obtained for MM (fig. S5, G to I) and pGBM samples (fig. S8). The CNV^−^ cells often appeared in multiple scATAC clusters, suggesting the presence of multiple distinct nonneoplastic cell clusters. To validate our cell assignments with an orthogonal method, we performed differential DNA recognition motif analysis for individual cell populations identified by Copy-scAT to look for differentially accessible transcription factor (TF) motifs associated with lineage specification. Differential motif analysis with ChromVAR confirmed high scores for neural progenitor cell–associated motifs, including ASCL1 in CNV^+^ cells (Fig. 3G), while the putative nonneoplastic clusters showed increased occupancy at DNA recognition motifs associated with TFs associated with the hematopoietic lineages, such as IKZF1 (Fig. 3H). Another CNV^−^ cluster showed enrichment of FOXG1 binding motifs in accessible chromatin, in keeping with a nonneoplastic neural cell identity (Fig. 3I). Using this approach, it was possible to discriminate between malignant and microenvironmental cells in all tumor samples analyzed (figs. S9 to S11). Clustering based on motif analysis (such as by ChromVAR) is another method for potentially distinguishing tumor from nontumor cells, and we wondered whether this would be sufficient for cell assignment. We therefore performed ChromVAR analysis followed by *k*-means clustering on our aGBM scATAC samples and compared the results to those obtained by Copy-scAT (fig. S12). While clustering based on motifs clearly delineated the more divergent hematopoietic infiltrating cells from the nontumor cells, this method was unable to distinguish between nonneoplastic brain cell types and GBM tumor cells, which have more similar accessibility profiles (fig. S12). Moreover, motif analysis is also dependent on defining a priori the motifs that would distinguish a neoplastic from a nonneoplastic cell type, which is a nontrivial task especially for GBM. Copy-scAT therefore is a useful approach to distinguish malignant from nonmalignant cells and to infer lineage relationships between genetic subclones that coexist in a tumor.

### Subclonal genetics is associated with chromatin accessibility profiles in aGBM

**Fig. 4.**
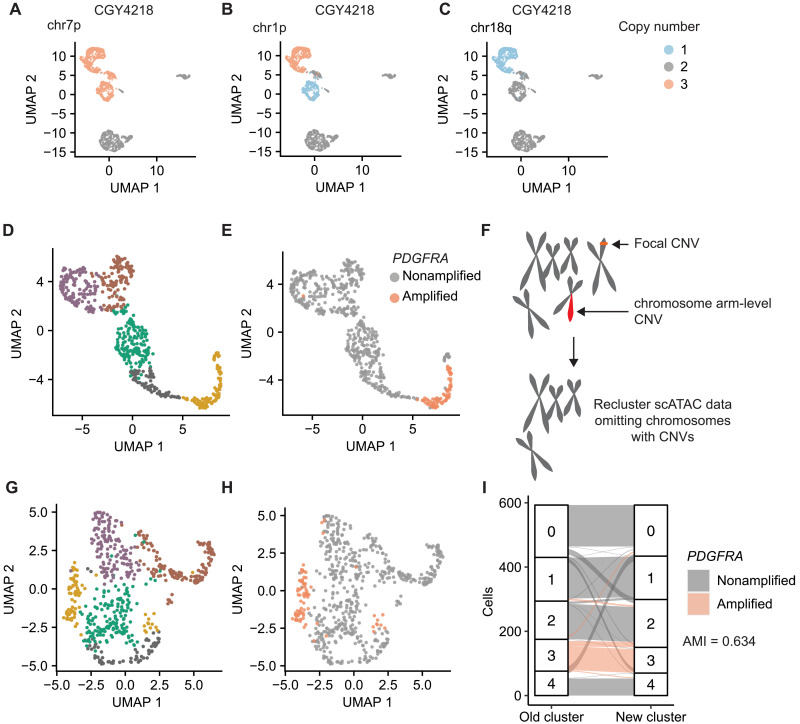
Subclonal genetics influences clustering of scATAC-seq data. (**A** to **C**) CNVs in aGBM CGY4218 segregate within specific scATAC clusters. (**D** and **E**) *PDGFRA*-amplified cells cluster together in aGBM CGY4349. (**F**) Diagram summarizing our strategy to remove CNVs from clustering of scATAC data. All chromosomes or regions with putative CNVs were removed from downstream analyses, and cells were reclustered. (**G**) Reclustering of (D) following removal of chromosomes and regions affected by CNVs in CGY4349. (**H**) Distribution of *PDGFRA*-amplified cells following reclustering. (**I**) Cluster assignments of cells in CGY4349 (aGBM specimen) before and after removal of CNV-containing regions (purple, *PDGFRA*-amplified cells).

We noticed that in most tumors that we analyzed, cells harboring a given CNV had a tendency to cluster together (Fig. 3, A to F). Individual clusters were defined by the presence of specific CNVs (Fig. 4, A to C). This was an unexpected observation that made us question whether clustering of scATAC data reflects the global patterns of chromatin accessibility. One possible explanation for this observation could be that chromosomal regions affected by a CNV display imbalances in the fragment depth distribution of scATAC datasets and that these patterns have a dominant effect on cluster assignment. Most scATAC-seq workflows rely on some variant of term frequency–inverse document frequency (TF-IDF) normalization rather than feature scaling, and this may amplify the effects of CNV-driven DNA content imbalances. For instance, it is possible that focal amplifications of the *PDGFRA* locus result in increased frequency of transposition events that are mapped to this site. A dominant effect of chromatin accessibility at this amplified locus could result in *PDGFRA*-amplified cells clustering together in UMAP representations of scATAC data (Fig. 4, D and E). We found that compared to a random selection of peaks, the chromosomes that carried CNVs had significantly different numbers of peaks ranked as highly variant than chromosomes that did not have CNVs, leading to a markedly uneven distribution of top peaks (*P* < 2.2 × 10^–16^, chi-square test; fig. S13). This was not seen in putative nonneoplastic cells, which had relatively even differentially accessible fragment distribution patterns (*P* = 0.05472, chi-square test; fig. S13). To test whether CNVs affect the clustering of scATAC data points, we removed all peaks mapping to chromosomes predicted to harbor CNVs by Copy-scAT and, lastly, reclustered all cells in each sample (Fig. 4F). We found that although removing chromosomes with CNVs from our analyses changed the overall cluster structure of a sample (Fig. 4G), *PDGFRA*-amplified cells still clustered close to each other (Fig. 4H). Our results indicate that clustering after CNV removal is more granular but overall stable, with moderate cluster concordance [adjusted mutual information (AMI) 0.634] (Fig. 4I). In this case, *PDGFRA*-amplified cells were localized to a single cluster before removing chromosomes affected by CNVs. Following removal of CNV^+^ chromosomes and reclustering, most *PDGFRA*-amplified cells still clustered together, with only a few cells merging into a cluster that included both amplified and nonamplified cells. Comparing the most variable peaks after chromosome CNV removal showed a distribution closer to normal, supporting the marked effect of the CNVs on the identification of variant peaks (*P* = 2.418 × 10^–8^, fig. S13C). These data indicate that genetic subclones may have characteristic patterns of chromatin accessibility and that a cell’s genetic background may influence its likelihood of attaining specific epigenetic states.

### Genetic events predispose subclones to the acquisition of developmental chromatin states

**Fig. 5.**
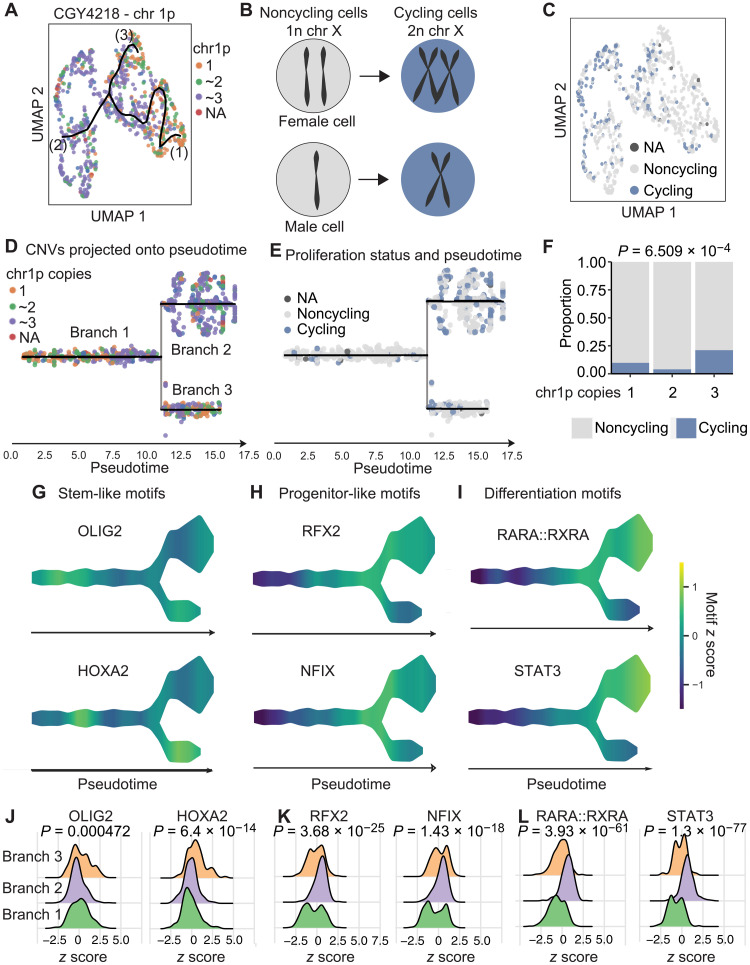
Subclonal genetic alterations predispose cells to adopt developmental chromatin states. (**A**) Cells were clustered on the basis of scATAC ChromVAR motif scores and then shaded on the basis of the presence of one, two, or three copies of chromosome 1p. NA, not available. (**B**) Schematic of method used to determine putative cycling cells. (**C**) Cells were shaded on the basis of their predicted cycling properties. (**D**) Data shown in (A) projected onto pseudotime. The resulting three branches are populated preferentially by cells with gain or loss of chromosome 1p, respectively. (**E**) Proliferation status as shown in (B), overlaid onto pseudotime. (**F**) Tumor cells with chromosome 1p gain show greater proportions of proliferative cells (statistics, chi-square test). (**G**) Scaled chromatin accessibility at binding motifs for OLIG2 and HOXA2, two TFs associated with stemness. (**H**) Scaled chromatin accessibility at binding motifs for RFX2 and NFIX, two TFs associated with progenitor-like phenotypes. (**I**) Scaled chromatin accessibility at binding motifs for RARA::RXRA and signal transducers and activators of transcription 3 (STAT3), two TFs associated with differentiated phenotypes. (**J**) Enrichment plot for motif *z* scores for OLIG2 and HOXA2. (**K**) Enrichment plot for motif *z* scores for RFX and NFIX. (**L**) Enrichment plot for motif *z* scores for RARA::RXRA and STAT3. *P* values were calculated by Kruskal-Wallis test.

We further explored the notion that CNVs may shape chromatin accessibility profiles and its possible implications for cell fate determination. As an illustrative example, we focused on an aGBM sample (CGY4218) where CNVs at chromosome 1p characterized three genetic subclones, as determined with Copy-scAT: (i) a subclone with two copies of chromosome 1p, (ii) a subclone with loss of 1p, and (iii) a subclone with gain of 1p (Fig. 5A).

We were interested in determining whether the major genetic subclones in this tumor had similar cycling properties. Unlike scRNA-seq, we found that it is not possible to use scATAC profiles at cell cycle genes to determine whether a cell is proliferating. We reasoned that cells that are actively going through cell division have to replicate their DNA. Given that cancer cells have numerous CNVs on autosomes and could lead to noisy data, we decided to use Copy-scAT to identify cells that have doubled the number of their X chromosomes and defined them as actively cycling cells (Fig. 5B). To validate this approach, we determined the number of cells with double the number of expected X chromosomes—i.e., putative cycling cells—in previously published scATAC datasets for mouse brain and peripheral blood mononuclear cells (PBMCs). We hypothesized that we should be able to identify cycling cells in fetal mouse brain but not in PBMCs. We detected numerous cycling cells (with twice the expected number of X chromosomes) in fetal mouse brain but not in PBMCs (fig. S14). Actively dividing cells can therefore be identified by inferring doubling of X chromosome number from baseline using scATAC data. The inferred cyling versus noncycling status was overlaid onto the UMAP plot, as shown above (Fig. 5C). We used scATAC data to arrange cells from this tumor along pseudotime with the package STREAM (Fig. 5D) (*14*) and then superimposed cell cycle status determined with our X chromosome doubling method (Fig. 5E). The results show that cells along branch 2, which is strongly enriched for cells with chromosome 1p gains, are also the most proliferative (Fig. 5, D and E. When we quantify the number of cycling cells grouped based on their 1p status, we see an enrichment in cycling cells in the 1p gain subclone, with approximately 25% of cells actively cycling, in comparison to less than 10% of those with 1p loss and less than 2% of those with two copies of 1p (*P* = 6.509 × 10^−4^, chi-square test; Fig. 5F and fig. S15). Similar results were noted when the data were analyzed by STREAM branch, with an enrichment of cycling cells in branch 2 (approximately 25% cycling cells; *P* = 7.776 × 10^−14^, chi-square test; fig. S15). These data therefore indicate functional differences between cells with gain or loss of chromosome 1p.

We then used ChromVAR (*15*) and STREAM-ATAC to calculate scores for TF binding motifs that are associated with neurodevelopmental processes. This analysis revealed that motifs bound by TFs that are associated with stem-like phenotypes, including OLIG2 and HOXA2, are enriched in accessible chromatin regions in cells that have one copy of chromosome 1p (Fig. 5G). Motifs bound by TFs associated with progenitor (Fig. 5H) and differentiated states (Fig. 5I) were enriched in the branch with more cells showing gain of chromosome 1p. This was associated with a significant shift in the overall distribution of enrichment of these motifs in cells along the different branches of the trajectory (Fig. 5, J to L). A distribution of genetic subclones along developmental chromatin accessibility states was observed in other tumor samples that we studied (figs. S16 to S18). Overall, the data support the notion that tumor cells may sample a discrete number of chromatin states, but their transition probabilities differ based on genotype. Consequently, chromatin states associated with each genetic subclone could manifest as different functional properties, here demonstrated at the level of cell proliferation and stemness profiles. The use of Copy-scAT therefore allows testing of hypotheses on the association between subclonal CNVs and cell behavior, which is relevant for many cancer types.

To test whether the association between CNVs and chromatin profiles extended beyond chromosome 1p status, we investigated other genetic subclones. We focused on subclones that harbored *EGFR*- or *PDGFRA*-amplified cells because these focal amplifications are mutually exclusive and therefore constitute well-defined subclonal compartments, as shown above (Fig. 3, E and F). We found that epigenetic differences within *EGFR-*amplified cells and *PDGFRA*-amplified cells in one of our aGBM samples extended beyond the amplified segments and imparted distinct global epigenetic profiles. When we examined differentially enriched TF motifs in accessible chromatin in CGY4349 cells that were amplified for *EGFR* and *PDGFRA*, we found that, as described previously (*5*), the *PDGFRA*-amplified cells showed increased occupancy at proneural motifs such as OLIG2, ASCL1, and NEUROD1, while the *EGFR*-amplified cells exhibited enrichment for motifs of the RFX family and the AP-1 family (FOS and JUN) (table S3). Together, our results illustrate the principle that genetic subclones can have unique accessible chromatin profiles that are associated with downstream functional properties.

## DISCUSSION

Here, we describe Copy-scAT, a computational tool dedicated to inferring CNVs using scATAC data. Copy-scAT resolves a computational bottleneck that has restricted the application of single-cell epigenomic techniques to the study of clinical tumor samples, which are often mixtures of malignant and nonmalignant cells. The presence of nonmalignant cells can severely confound the analyses of these samples and downstream data interpretation. Cell admixture is a particular problem for scATAC data because of the inherent sparsity of these datasets and because they do not provide direct information on the expression status of cell lineage markers that could be used to solve cellular identities. For tumor types that harbor CNVs, Copy-scAT provides a simple way of solving this problem. However, a limitation of Copy-scAT is that it cannot be used with malignancies that are driven primarily by single nucleotide variants and have no or rare CNVs.

It is important to note that Copy-scAT enables users to perform analyses on both malignant and nonmalignant cells from a tumor sample, because cell barcodes associated with either the presence or absence of CNVs can be selected for downstream analyses. Implementation of Copy-scAT will therefore be beneficial to groups interested in defining the epigenomes of both tumor cells and their microenvironment. Because chromatin accessibility datasets provide information on mechanisms of transcriptional regulation by distal and proximal enhancer and super enhancer elements, Copy-scAT could be useful in clarifying epigenetic mechanisms involved in tumor immune suppression and T cell exhaustion, for instance. Copy-scAT also allows scATAC studies of unsorted frozen banked cancer specimens (see Materials and Methods), because it requires no prior knowledge of cell type composition.

We show that the underlying CNV architecture of tumor cells plays a significant role in clustering of scATAC data, a problem that can be amplified by the use of TF-IDF algorithms for normalization. These effects are less pronounced when clustering is based on motif activity scores (e.g., ChromVAR), likely as this incorporates data from multiple chromosomes, thus dampening the effect of variation at any one specific locus. Further studies are needed to identify the optimal way to address the effects of CNVs in downstream analyses of scATAC datasets as CNVs may represent a significant confounder and potentially mask significant biological relationships.

Copy-scAT can be used to shed new light on how genetics and epigenetics interface in cancer. Our data expand on previous reports of parallel evolution of genetic and DNA methylation states in gliomas (*16*). Here, we show that genetic subclones tend to have unique chromatin accessibility landscapes that are associated with differential DNA recognition motif usage and downstream functional properties. Consequently, some genetic subclones have greater proportions of stem-like cells, and others appear more differentiated and have diverse proliferative capacities. This is likely because specific genetic alterations (for instance, focal amplification of *EGFR* or *PGDFRA*) could result in downstream increased activity of specific TFs, which then contribute to the transcriptional output of that subclone. Our findings complement the observed high levels of intratumoral transcriptional heterogeneity in GBM (*5*, *17*) by suggesting that subclonal genetic alterations predispose a cell to populating particular gene regulatory states. This is in keeping with recent transcriptomics studies that have suggested that in IDH wild-type GBM, the presence of specific genetic alterations is associated with defined transcriptional states (*5*, *18*). Future work will be required to determine whether CNVs (i) prime cells to acquire specific chromatin accessibility states or (ii) have roles in stabilizing chromatin states of genetic subclones.

Copy-scAT will enable future studies of subclonal chromatin dynamics in complex tumor types and may be an important tool to better understand the functional relationships between subclones, their microenvironment, and therapy response.

## MATERIALS AND METHODS

### Ethics and consent statement

All samples were collected and used for research with appropriate informed consent and with approval by the Health Research Ethics Board of Alberta.

### scATAC-seq sample processing

GBM samples were either frozen surgical resections (pGBM) or cells dissociated from fresh surgical specimens and cryopreserved (aGBM). Samples were dissociated in a 1.5-ml microcentrfuge tube, using a wide-bore P1000 pipette followed by a narrow bore P1000 pipette in nuclear resuspension buffer [10 mM tris-HCl, 10 mM NaCl, 3 mM MgCl_2_, 0.1% IGEPAL, 0.1% Tween 20, 0.01% digitonin, and 1% bovine serum albumin (BSA) in phosphate-buffered saline (PBS)], then vortexed briefly, chilled on ice for 10 min, then pipetted again, and spun at 4°C at 500*g* for 5 min. This step was repeated, and the sample was then resuspended in Tween 20 wash buffer (10 mM tris-HCl, 10 mM NaCl, 3 mM MgCl_2_, 0.1% IGEPAL, 0.1% Tween 20, and 1% BSA in PBS) and then strained through a 35-μm cell strainer fluorescence-activated cell sorting tube (Thermo Fisher Scientific, 08-771-23) to remove debris. Nuclei were then quantified by trypan blue on the Countess II (Invitrogen), spun down at 500*g* at 4°C for 5 min, and resuspended in the nuclear isolation buffer (10x Genomics), and the rest of the scATAC was performed as per the 10x Genomics protocol. MM samples were from bone marrow aspirates collected from patients; tumor cells were isolated from mononuclear cell fractions through Ficoll gradients coupled with magnetic bead sorting of CD138^+^ cells. scATAC libraries were prepared from GBM and MM samples using a Chromium Controller (10x Genomics). Libraries were sequenced on NextSeq 500 or Novaseq 6000 instruments (Illumina) at the Centre for Health Genomics and Informatics (CHGI; University of Calgary) using the recommended settings.

### scATAC-seq initial data analysis

The raw sequencing data were demultiplexed using cellranger-atac mkfastq (Cell Ranger ATAC, version 1.1.0, 10x Genomics). scATAC-seq reads were aligned to the hg38 reference genome (GRCh38, version 1.1.0, 10x Genomics) and quantified using cellranger-atac count function with default parameters (Cell Ranger ATAC, version 1.1.0, 10x Genomics).

### scCNV analysis

#### Fragment pileup and normalization

The fragment file was processed, and signal was binned into bins of a preset size (default 1 Mb) across the hg38 chromosomes to generate a genome-wide read-depth map. Only barcodes with a minimum of 5000 reads were retained, to remove spurious barcodes. This flattened barcode-fragment matrix pileup was cleaned by removal of genomic intervals that were uninformative (greater than 80% zeros) and barcodes with greater than a certain number of zero intervals. Cells passing this first filter were normalized with counts per million (cpm) normalization using cpm in the edgeR package (*19*).

#### Chromosome arm CNV analysis

The normalized barcode-fragment matrix was collapsed to the chromosome arm level, using chromosome arm information from the UCSC University of California Santa Cruz (UCSC table: cytoBand), centromeres were removed, and signal in each bin was normalized using the number of base pairs in CpG islands in the interval using the UCSC CpG islands table (UCSC table: cpgIslandExtUnmasked). The signal was then summarized using a quantile-trimmed-mean (between the 50th and 80th quantiles). Only chromosome arms with a minimum trimmed mean signal were kept for analysis.

The chromosome arm signal matrix is mixed with a generated set proportion of pseudodiploid control cells, defined using the mean of chromosome segment medians with a defined SD. This cell-signal matrix is then scaled across each chromosome arm and centered on the median signal of all chromosomes. Each chromosome arm segment is then analyzed using Gaussian decomposition with Mclust (*20*). The subsequent clusters are filtered on the basis of *z* scores and mixing proportions, and redundant clusters are combined. These *z* scores are then translated into estimated copy numbers for each segment for each barcode. The barcode CNV assignments can be optionally used to assign consensus CNVs to clusters generated in other software packages such as Loupe or Seurat/Signac.

Clusters for smoothing were generated by loading the data into Signac 1.0.0 (*21*). Datasets were quality filtered, keeping cells containing at least 3000 peak region fragments, greater than 15% of reads in peaks, and lowmapq < 30,000. These were then normalized using TF-IDF, and latent semantic analysis was used for dimensionality reduction by SVD (singular value decomposition), followed by UMAP using dimensions 2:50, and nearest-neighbor clustering (using dimensions 2:50 and *k* value of 21). Smoothing was performed using Copy-scAT with a boost value of 0 (taking an average of the imputed chromosome value for the cluster followed by rounding to get a consensus value).

#### Detection of amplifications

The normalized barcode-fragment matrix was scaled, and mean-variance changepoint analysis using the changepoint package was performed for each cell and each chromosome to identify areas of abnormally high signal (*z* score greater than 5) (*22*). The consensus coordinates of each amplification region were generated across all cells, and only abnormalities affecting a minimum number of cells were kept for analysis.

### scATAC trajectory analysis

STREAM-ATAC and STREAM (*23*) were used to generate pseudotime trajectories on the basis of motif occupancy profiles generated using ChromVAR (*24*) with the JASPAR 2018 motif database (*25*). Dimensionality reduction was performed using the top 20 components and 50 neighbors, and an initial elastic graph was generated on the two-dimensional UMAP projection using 10 clusters, using the *K*-means method with n_neighbors = 30. An elastic principal graph was constructed using the parameters epg_alpha = 0.02, epg_mu = 0.05, epg_lambda = 0.02, and epg_trimmingradius = 1.2, with branch extension using “QuantDists.” Trees were rooted using the branch with highest motif activities for OLIG2 and ETV motifs as root.

### Whole-genome sequencing

DNA was extracted from residual nuclei from the same samples and tissue fragments used for scATAC-seq of aGBM samples using the Qiagen DNEasy Blood and Tissue DNA Extraction Kit (QIAGEN, no. 69504). Libraries were prepared using the NEBNext Ultra II DNA Library Prep Kit (no. E7645) and sequenced on the Novaseq 6000 (Illumina) at the CHGI (University of Calgary) in paired-end mode.

### Whole-genome data processing

Genome data were aligned to the hg38 assembly using bwa mem (bwa 0.7.17) (*26*). SAMtools was used to extract high-quality reads (*Q* > 30), and Picard tools (Broad Institute) was used to remove duplicates (*27*).

### Whole-genome SNV and CNV detection

Gatk mutect2 (Broad Institute) was run on the filtered data to detect SNVs with low stringency using the following settings: --disable-read-filter MateOnSameContigOrNoMappedMateReadFilter. CNVkit was subsequently used to call CNVs using the following parameters: --filter cn -m clonal –purity 0.7 (*28*). Adjacent segments were further combined and averaged using bedtools (*29*).

### Data visualization and clustering

Data were visualized, and UMAP plots were generated using Seurat 3.0.0 and Signac 1.0.0 (*21*, *30*) and Cell Loupe version 4.0.0.

### Statistical analysis

Between-group differences in discrete values (e.g., chromosome peaks and branch assignments) were calculated using the chi-square test. Differences in nonparametric distributions (motif accessibility in clusters) were quantified using the Kruskal-Wallis test.

## Notes

View/request a protocol for this paper from *Bio-protocol*.

## References

[1] B. Lim, Y. Lin, N. Navin, Advancing cancer research and medicine with single-cell genomics. Cancer Cell 37, 456–470 (2020). 32289270 10.1016/j.ccell.2020.03.008 PMC7899145

[2] A. P. Patel, I. Tirosh, J. J. Trombetta, A. K. Shalek, S. M. Gillespie, H. Wakimoto, D. P. Cahill, B. V. Nahed, W. T. Curry, R. L. Martuza, D. N. Louis, O. Rozenblatt-Rosen, M. L. Suvà, A. Regev, B. E. Bernstein, Single-cell RNA-seq highlights intratumoral heterogeneity in primary glioblastoma. Science 344, 1396–1401 (2014). 24925914 10.1126/science.1254257 PMC4123637

[3] S. Darmanis, S. A. Sloan, D. Croote, M. Mignardi, S. Chernikova, P. Samghababi, Y. Zhang, N. Neff, M. Kowarsky, C. Caneda, G. Li, S. D. Chang, I. D. Connolly, Y. Li, B. A. Barres, M. H. Gephart, S. R. Quake, Single-Cell RNA-Seq analysis of infiltrating neoplastic cells at the migrating front of human glioblastoma. Cell Rep. 1399–1410 (2017). 29091775 10.1016/j.celrep.2017.10.030 PMC5810554

[4] J. Gojo, B. Englinger, L. Jiang, J. M. Hübner, M. L. Shaw, O. A. Hack, S. Madlener, D. Kirchhofer, I. Liu, J. Pyrdol, V. Hovestadt, E. Mazzola, N. D. Mathewson, M. Trissal, D. Lötsch, C. Dorfer, C. Haberler, A. Halfmann, L. Mayr, A. Peyrl, R. Geyeregger, B. Schwalm, M. Mauermann, K. W. Pajtler, T. Milde, M. E. Shore, J. E. Geduldig, K. Pelton, T. Czech, O. Ashenberg, K. W. Wucherpfennig, O. Rozenblatt-Rosen, S. Alexandrescu, K. L. Ligon, S. M. Pfister, A. Regev, I. Slavc, W. Berger, M. L. Suvà, M. Kool, M. G. Filbin, Single-cell RNA-seq reveals cellular hierarchies and impaired developmental trajectories in pediatric ependymoma. Cancer Cell 38, 44–59 (2020). 32663469 10.1016/j.ccell.2020.06.004 PMC7479515

[5] C. Neftel, J. Laffy, M. G. Filbin, T. Hara, M. E. Shore, G. J. Rahme, A. R. Richman, D. Silverbush, M. L. Shaw, C. M. Hebert, J. Dewitt, S. Gritsch, E. M. Perez, L. N. Gonzalez Castro, X. Lan, N. Druck, C. Rodman, D. Dionne, A. Kaplan, M. S. Bertalan, J. Small, K. Pelton, S. Becker, D. Bonal, Q.-D. Nguyen, R. L. Servis, J. M. Fung, R. Mylvaganam, L. Mayr, J. Gojo, C. Haberler, R. Geyeregger, T. Czech, I. Slavc, B. V. Nahed, W. T. Curry, B. S. Carter, H. Wakimoto, P. K. Brastianos, T. T. Batchelor, A. Stemmer-Rachamimov, M. Martinez-Lage, M. P. Frosch, I. Stamenkovic, N. Riggi, E. Rheinbay, M. Monje, O. Rozenblatt-Rosen, D. P. Cahill, A. P. Patel, T. Hunter, I. M. Verma, K. L. Ligon, D. N. Louis, A. Regev, B. E. Bernstein, I. Tirosh, M. L. Suvà, An integrative model of cellular states, plasticity, and genetics for glioblastoma. Cell 178, 835–849.e21 (2019). 31327527 10.1016/j.cell.2019.06.024 PMC6703186

[6] M. C. Vladoiu, I. El-Hamamy, L. K. Donovan, H. Farooq, B. L. Holgado, Y. Sundaravadanam, V. Ramaswamy, L. D. Hendrikse, S. Kumar, S. C. Mack, J. J. Y. Lee, V. Fong, K. Juraschka, D. Przelicki, A. Michealraj, P. Skowron, B. Luu, H. Suzuki, A. S. Morrissy, F. M. G. Cavalli, L. Garzia, C. Daniels, X. Wu, M. A. Qazi, S. K. Singh, J. A. Chan, M. A. Marra, D. Malkin, P. Dirks, L. Heisler, T. Pugh, K. Ng, F. Notta, E. M. Thompson, C. L. Kleinman, A. L. Joyner, N. Jabado, L. Stein, M. D. Taylor, Childhood cerebellar tumours mirror conserved fetal transcriptional programs. Nature 572, 67–73 (2019). 31043743 10.1038/s41586-019-1158-7 PMC6675628

[7] I. Tirosh, A. S. Venteicher, C. Hebert, L. E. Escalante, A. P. Patel, K. Yizhak, J. M. Fisher, C. Rodman, C. Mount, M. G. Filbin, C. Neftel, N. Desai, J. Nyman, B. Izar, C. C. Luo, J. M. Francis, A. A. Patel, M. L. Onozato, N. Riggi, K. J. Livak, D. Gennert, R. Satija, B. V. Nahed, W. T. Curry, R. L. Martuza, R. Mylvaganam, A. J. Iafrate, M. P. Frosch, T. R. Golub, M. N. Rivera, G. Getz, O. Rozenblatt-Rosen, D. P. Cahill, M. Monje, B. E. Bernstein, D. N. Louis, A. Regev, M. L. Suvà, Single-cell RNA-seq supports a developmental hierarchy in human oligodendroglioma. Nature 539, 309–313 (2016). 27806376 10.1038/nature20123 PMC5465819

[8] A. S. Venteicher, I. Tirosh, C. Hebert, K. Yizhak, C. Neftel, M. G. Filbin, V. Hovestadt, L. E. Escalante, M. L. Shaw, C. Rodman, S. M. Gillespie, D. Dionne, C. C. Luo, H. Ravichandran, R. Mylvaganam, C. Mount, M. L. Onozato, B. V. Nahed, H. Wakimoto, W. T. Curry, A. J. Iafrate, M. N. Rivera, M. P. Frosch, T. R. Golub, P. K. Brastianos, G. Getz, A. P. Patel, M. Monje, D. P. Cahill, O. Rozenblatt-Rosen, D. N. Louis, B. E. Bernstein, A. Regev, M. L. Suvà, Decoupling genetics, lineages, and microenvironment in IDH-mutant gliomas by single-cell RNA-seq. Science 355, eaai8478 (2017). 28360267 10.1126/science.aai8478 PMC5519096

[9] S. Müller, A. Cho, S. J. Liu, D. A. Lim, A. Diaz, CONICS integrates scRNA-seq with DNA sequencing to map gene expression to tumor sub-clones. Bioinformatics 34, 3217–3219 (2018). 29897414 10.1093/bioinformatics/bty316 PMC7190654

[10] J. D. Buenrostro, P. G. Giresi, L. C. Zaba, H. Y. Chang, W. J. Greenleaf, Transposition of native chromatin for fast and sensitive epigenomic profiling of open chromatin, DNA-binding proteins and nucleosome position. Nat. Methods 10, 1213–1218 (2013). 24097267 10.1038/nmeth.2688 PMC3959825

[11] J. D. Buenrostro, B. Wu, U. M. Litzenburger, D. Ruff, M. L. Gonzales, M. P. Snyder, H. Y. Chang, W. J. Greenleaf, Single-cell chromatin accessibility reveals principles of regulatory variation. Nature 523, 486–490 (2015). 26083756 10.1038/nature14590 PMC4685948

[12] R. Killick, I. A. Eckley, Changepoint: An R package for changepoint analysis. *J. Stat. Softw.* (2014), doi:10.18637/jss.v058.i03.

[13] M. Snuderl, L. Fazlollahi, L. P. Le, M. Nitta, B. H. Zhelyazkova, C. J. Davidson, S. Akhavanfard, D. P. Cahill, K. D. Aldape, R. A. Betensky, D. N. Louis, A. J. Iafrate, Mosaic amplification of multiple receptor tyrosine kinase genes in glioblastoma. *Cancer Cell* (2011), doi:10.1016/j.ccr.2011.11.005. 10.1016/j.ccr.2011.11.005 22137795

[14] H. Chen, L. Albergante, J. Y. Hsu, C. A. Lareau, G. Lo Bosco, J. Guan, S. Zhou, A. N. Gorban, D. E. Bauer, M. J. Aryee, D. M. Langenau, A. Zinovyev, J. D. Buenrostro, G. C. Yuan, L. Pinello, Single-cell trajectories reconstruction, exploration and mapping of omics data with STREAM. Nat. Commun. 10, 1903 (2019). 31015418 10.1038/s41467-019-09670-4 PMC6478907

[15] A. N. Schep, B. Wu, J. D. Buenrostro, W. J. Greenleaf, ChromVAR: Inferring transcription-factor-associated accessibility from single-cell epigenomic data. Nat. Methods 14, 975–978 (2017). 28825706 10.1038/nmeth.4401 PMC5623146

[16] T. Mazor, A. Pankov, B. E. Johnson, C. Hong, E. G. Hamilton, R. J. A. Bell, I. V. Smirnov, G. F. Reis, J. J. Phillips, M. J. Barnes, A. Idbaih, A. Alentorn, J. J. Kloezeman, M. L. M. Lamfers, A. W. Bollen, B. S. Taylor, A. M. Molinaro, A. B. Olshen, S. M. Chang, J. S. Song, J. F. Costello, DNA Methylation and Somatic Mutations Converge on the Cell Cycle and Define Similar Evolutionary Histories in Brain Tumors. *Cancer Cell* (2015), doi:10.1016/j.ccell.2015.07.012. 10.1016/j.ccell.2015.07.012 PMC4573399 26373278

[17] A. P. Patel, I. Tirosh, J. J. Trombetta, A. K. Shalek, S. M. Gillespie, H. Wakimoto, D. P. Cahill, B. V. Nahed, W. T. Curry, R. L. Martuza, D. N. Louis, O. Rozenblatt-Rosen, M. L. Suvà, A. Regev, B. E. Bernstein, Single-cell RNA-seq highlights intratumoral heterogeneity in primary glioblastoma. Science 344, 1396–1401 (2014). 24925914 10.1126/science.1254257 PMC4123637

[18] K. C. Johnson, K. J. Anderson, E. T. Courtois, F. P. Barthel, F. S. Varn, D. Luo, M. Seignon, E. Yi, H. Kim, M. R. H. Estecio, M. Tang, N. E. Navin, R. Maurya, C. Y. Ngan, N. Verburg, P. C. de Witt Hamer, K. Bulsara, M. L. Samuels, S. Das, P. Robson, R. G. W. Verhaak, Single-cell multimodal glioma analyses reveal epigenetic regulators of cellular plasticity and environmental stress response. *bioRxiv* (2020), doi:10.1101/2020.07.22.215335.

[19] M. D. Robinson, D. J. McCarthy, G. K. Smyth, edgeR: A bioconductor package for differential expression analysis of digital gene expression data. Bioinformatics 26, 139–140 (2010). 19910308 10.1093/bioinformatics/btp616 PMC2796818

[20] L. Scrucca, M. Fop, T. B. Murphy, A. E. Raftery, Mclust 5: Clustering, classification and density estimation using Gaussian finite mixture models. R J. 8, 289–317 (2016). 27818791 PMC5096736

[21] T. Stuart, A. Srivastava, C. Lareau, R. Satija, *bioRxiv*, in press, doi:10.1101/2020.11.09.373613.

[22] R. Killick, I. A. Eckley, Changepoint: An R package for changepoint analysis. *J. Stat. Softw.* **58** (2014), doi:10.18637/jss.v058.i03.

[23] H. Chen, L. Albergante, J. Y. Hsu, C. A. Lareau, G. Lo Bosco, J. Guan, S. Zhou, A. N. Gorban, D. E. Bauer, M. J. Aryee, D. M. Langenau, A. Zinovyev, J. D. Buenrostro, G. C. Yuan, L. Pinello, Single-cell trajectories reconstruction, exploration and mapping of omics data with STREAM. *Nat. Commun.* (2019), doi:10.1038/s41467-019-09670-4. 10.1038/s41467-019-09670-4 PMC6478907 31015418

[24] A. N. Schep, B. Wu, J. D. Buenrostro, W. J. Greenleaf, ChromVAR: Inferring transcription-factor-associated accessibility from single-cell epigenomic data. *Nat. Methods* (2017), doi:10.1038/nmeth.4401. 10.1038/nmeth.4401 PMC5623146 28825706

[25] A. Khan, O. Fornes, A. Stigliani, M. Gheorghe, J. A. Castro-Mondragon, R. Van Der Lee, A. Bessy, J. Chèneby, S. R. Kulkarni, G. Tan, D. Baranasic, D. J. Arenillas, A. Sandelin, K. Vandepoele, B. Lenhard, B. Ballester, W. W. Wasserman, F. Parcy, A. Mathelier, JASPAR 2018: Update of the open-access database of transcription factor binding profiles and its web framework. *Nucleic Acids Res.* (2018), doi:10.1093/nar/gkx1126. 10.1093/nar/gkx1126 PMC5753243 29140473

[26] H. Li, R. Durbin, Fast and accurate short read alignment with Burrows-Wheeler transform. Bioinformatics 25, 1754–1760 (2009). 19451168 10.1093/bioinformatics/btp324 PMC2705234

[27] H. Li, B. Handsaker, A. Wysoker, T. Fennell, J. Ruan, N. Homer, G. Marth, G. Abecasis, R. Durbin, The Sequence Alignment/Map format and SAMtools. Bioinformatics 25, 2078–2079 (2009). 19505943 10.1093/bioinformatics/btp352 PMC2723002

[28] E. Talevich, A. H. Shain, T. Botton, B. C. Bastian, CNVkit: Genome-Wide Copy Number Detection and Visualization from Targeted DNA Sequencing. *PLoS Comput. Biol.* **12** (2016), doi:10.1371/journal.pcbi.1004873. 10.1371/journal.pcbi.1004873 PMC4839673 27100738

[29] A. R. Quinlan, I. M. Hall, BEDTools: A flexible suite of utilities for comparing genomic features. Bioinformatics 26, 841–842 (2010). 20110278 10.1093/bioinformatics/btq033 PMC2832824

[30] A. Butler, P. Hoffman, P. Smibert, E. Papalexi, R. Satija, Integrating single-cell transcriptomic data across different conditions, technologies, and species. Nat. Biotechnol. 36, 411–420 (2018). 29608179 10.1038/nbt.4096 PMC6700744

[31] M. Hoffman, A. H. Gillmor, D. J. Kunz, M. J. Johnston, A. Nikolic, K. Narta, M. Zarrei, J. King, K. Ellestad, N. H. Dang, F. M. G. Cavalli, M. M. Kushida, F. J. Coutinho, Y. Zhu, B. Luu, Y. Ma, A. Mungall, R. Moore, M. A. Marra, M. D. Taylor, T. J. Pugh, P. B. Dirks, D. Strother, L. Lafay-Cousin, A. C. Resnick, S. Scherer, D. L. Senger, B. D. Simons, J. A. Chan, A. S. Morrissy, M. Gallo, Intratumoral genetic and functional heterogeneity in pediatric glioblastoma. Cancer Res. 79, 2111–2123 (2019). 30877103 10.1158/0008-5472.CAN-18-3441 PMC7282886

