# Supplementary material for: Copy-scAT: Deconvoluting single-cell chromatin accessibility of genetic subclones in cancer
Source: Sci Adv. 2021 Oct 13;7(42):eabg6045. doi: 10.1126/sciadv.abg6045 (PMC8514091; doi:10.1126/sciadv.abg6045)

Science Advances
AAAS

Supplementary Materials for

Copy-scAT: Deconvoluting single-cell chromatin accessibility of genetic subclones in cancer

Ana Nikolic, Divya Singhal, Katrina Ellestad, Michael Johnston, Yaoqing Shen, Aaron Gillmor, Sorana Morrissy, J. Gregory Cairncross, Steven Jones, Mathieu Lupien, Jennifer A. Chan, Paola Neri, Nizar Bahlis, Marco Gallo*

*Corresponding author. Email: marco.gallo@ucalgary.ca

Published 13 October 2021, *Sci. Adv.* **7**, eabg6045 (2021)
DOI: 10.1126/sciadv.abg6045

The PDF file includes:

Tables S1 and S2
Figs. S1 to S18

Other Supplementary Material for this manuscript includes the following:

Data file S1

SUPPLEMENTAL MATERIAL

Sample	Unique barcodes after pileup	Unique barcodes after filtering	Percent passing filters
CGY4218	1542	1289	83.59%
CGY4250	1371	1219	88.91%
CGY4275	1004	821	81.77%
CGY4349	961	807	83.98%
pCGY2932	1230	1025	83.33%
pCGY2937	1445	1200	83.04%
pCGY3402	3162	2625	83.02%
pCGY3749	2774	2473	89.15%
pCGY4021	1963	1623	82.68%
MM1217	936	847	90.49%
MM1388	2813	2506	89.09%
MM1389	7745	7133	92.10%
MM1438	1862	1721	92.43%
MM1460	2344	1943	82.89%
MM1479	5315	4564	85.87%
MM1498	7408	6324	85.37%
MM1555	7548	6743	89.33%
MM1643	4465	4168	93.35%
MM1698	3725	3092	83.01%
**Total cells profiled**	**59613**	**52123**	**86.49%**

Table S1. Summary of samples and cells profiled by Copy-scAT

Sample and Alteration	Precision	Recall	Accuracy
**Adult GBM (n = 3)**			
Gain	0.47	1.0	0.87
Loss	0.55	0.7	0.78
Amplification	1	1.0	1.0
**Pediatric GBM (n = 5)**			
Gain	0.56	0.88	0.83
Loss	0.72	0.66	0.92
**Multiple myeloma (n = 10)**			
Gain	0.57	0.77	0.82
Loss	0.43	0.75	0.72
**Overall (n = 18)**			
Gain	0.55	0.84	0.83
Loss	0.53	0.72	0.79
Amplification	1.0	1.0	1.0

Table S2. Precision, recall and accuracy of Copy-scAT in aGBM, pGBM and MM samples

Fig S1. Overview of changepoint analysis optimization with Copy-scAT. (A) Elbow plot of identified consensus amplification regions with different Z-score thresholds in one aGBM sample (CGY4250). **(B)** Number of cells flagged as containing amplifications at different Z score thresholds. **(C)** Example of changepoint regions identified using mean-based algorithm (Z-score values in black, regions in red). **(D)** Example of changepoint regions called by mean and variance-based changepoint (Z-score values in black, regions in red).

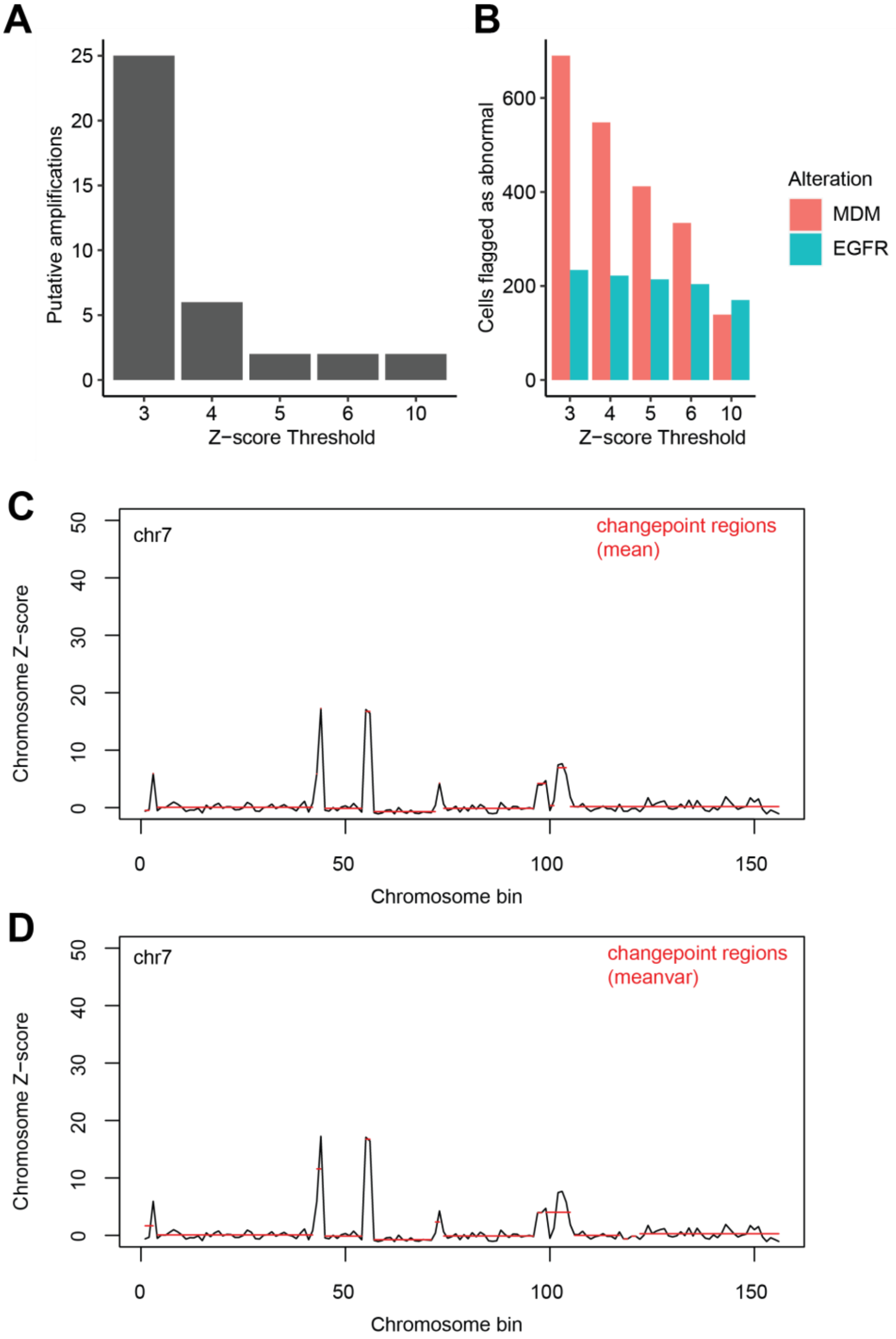

Fig. S2. Comparison of CNVs inferred by Copy-scAT and by WGS for adult GBM samples.

(A) Comparison of chromosome arm level losses detected in three adult GBM samples by single cell ATAC, WGS, or both methods. **(B)** Comparison of chromosome arm level losses detected in three adult GBM samples by single cell ATAC, WGS, or both methods. **(C)** Comparison of focal amplifications detected in three adult GBM sample by scATAC, WGS, or both methods.

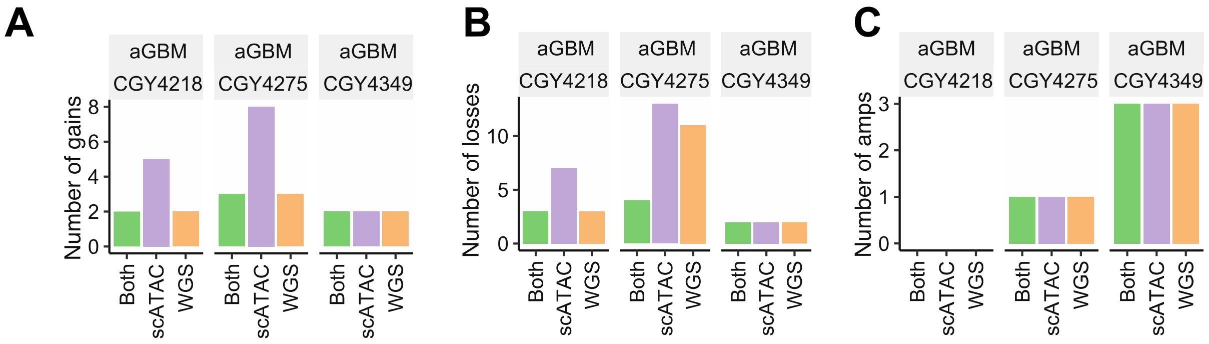

Fig. S3. Comparison of CNVs inferred by Copy-scAT or WGS in pediatric GBM samples. Overview of gains **(A)** and losses **(B)** detected in five pediatric samples by linked-reads WGS or single cell ATAC-seq.

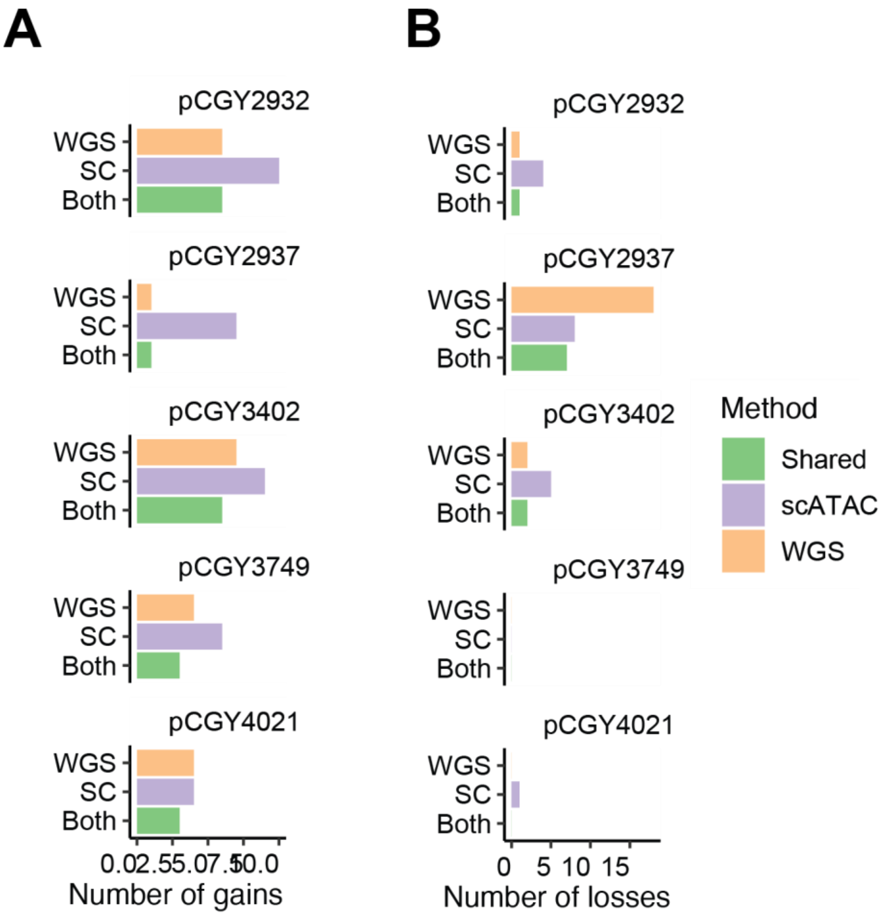

Fig. S4. Comparison of CNVs inferred by Copy-scAT or with the scCNV assay in multiple myeloma samples.
Overview of all gains **(A)** and losses **(B)** for each sample detected, by scATAC, scCNV, and both methods.

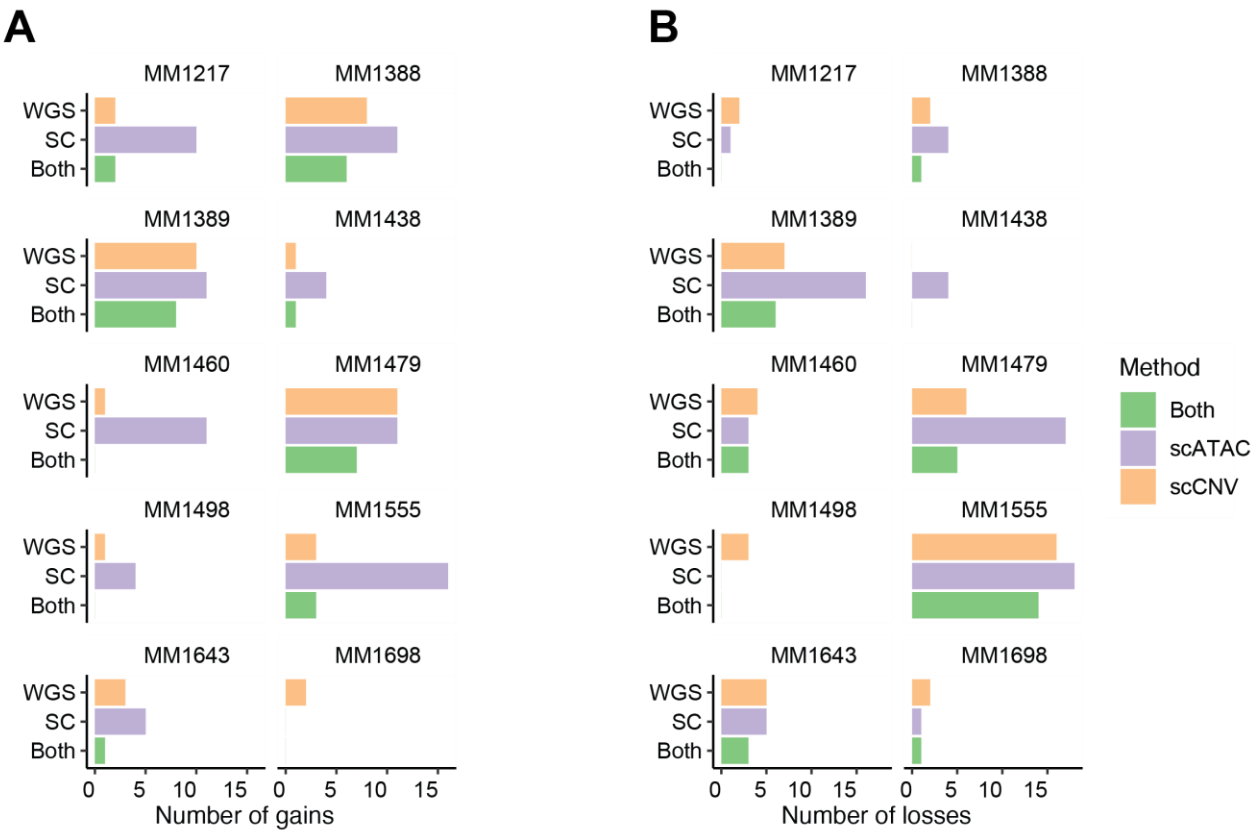

Fig. S5. Further comparisons of Copy-scAT results on myeloma samples.
(A-B) Proportion of chromosome arm-level copy number gains detected by both single-cell ATAC and 10X single-cell CNV analysis, plotted against the number of cells profiled by scATAC **(A)** and the average number of read fragments per cell in the scATAC sample **(B)**. **(C-D)** Proportion of chromosome arm-level copy number losses detected by both single-cell ATAC and 10X single-cell CNV analysis, plotted against the number of cells profiled by scATAC **(C)** and the average number of read fragments per cell in the scATAC sample **(D)**. **(E-F)** Comparison of number of distinct chromosomal arm-level gains **(E)** and losses **(F)** detected by either scATAC-seq (scATAC) or 10X scCNV analysis (scCN), plotted versus the number of cells profiled by scATAC. **(G-I)** UMAP showing distribution of chromosomal alterations (gains of chr11p **(G)** and chr11q **(H)**, loss of chr13q **(I)**) in myeloma sample MM1555.

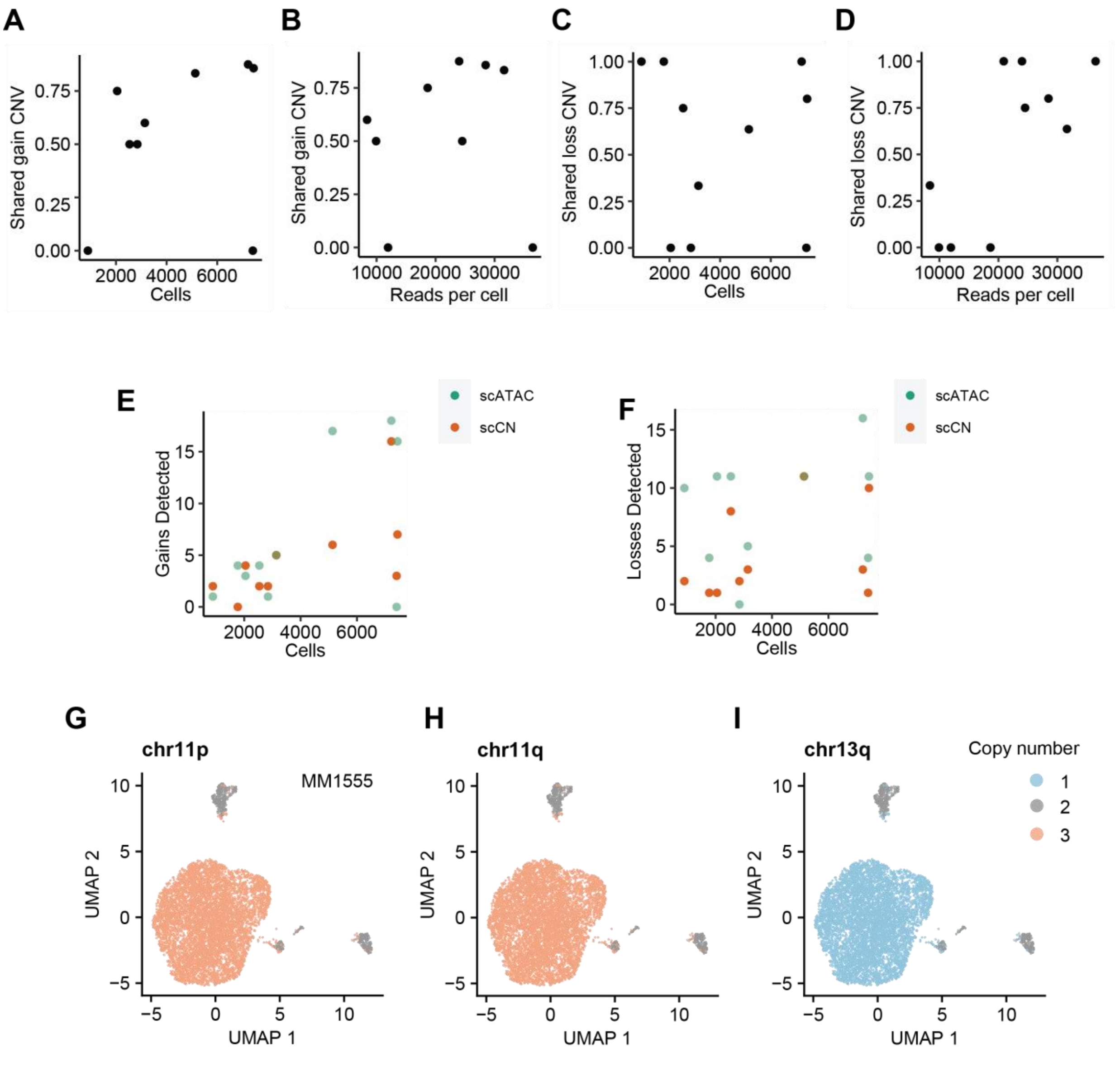

Fig. S6. Chromosome-level gains and losses in adult GBM samples before and after consensus cluster smoothing. (A) Overview of the four datasets included in the consensus UMAP. **(B)** Summary of CNV status concordance by cluster by chromosome arm for four adult GBM datasets before and after smoothing is applied (average 0.833; standard deviation 0.112). Chromosome 10q status before **(C)** and after **(D)** smoothing by consensus clustering. Chromosome 7p status before **(E)** and after **(F)** smoothing based on consensus clustering.

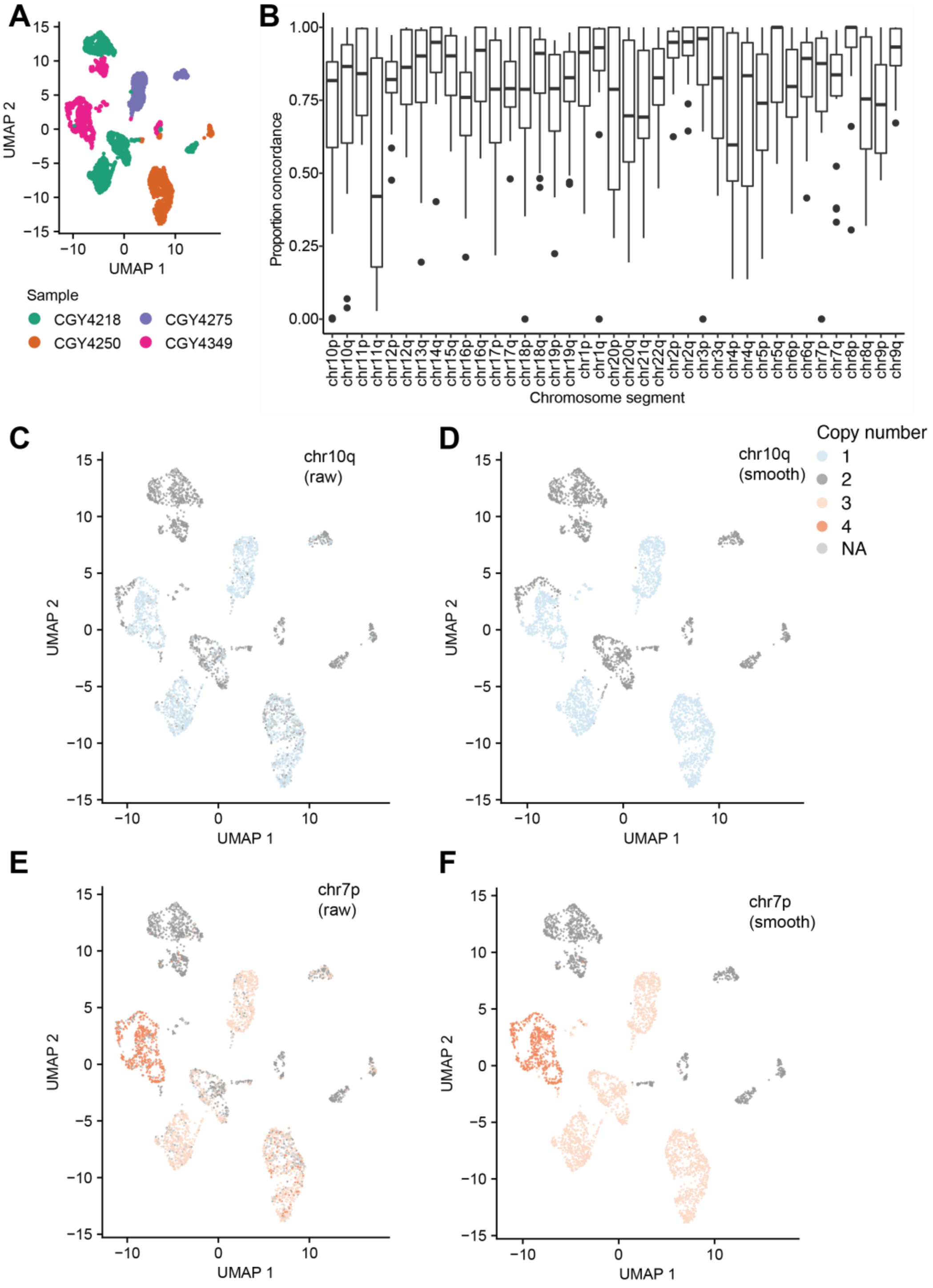

Fig. S7. Amplifications in adult GBM samples before and after consensus cluster smoothing. *PDGFRA* amplification before **(A)** and after **(B)** applying smoothing algorithm. *EGFR* amplification before **(C)** and after **(D)** applying smoothing algorithm.

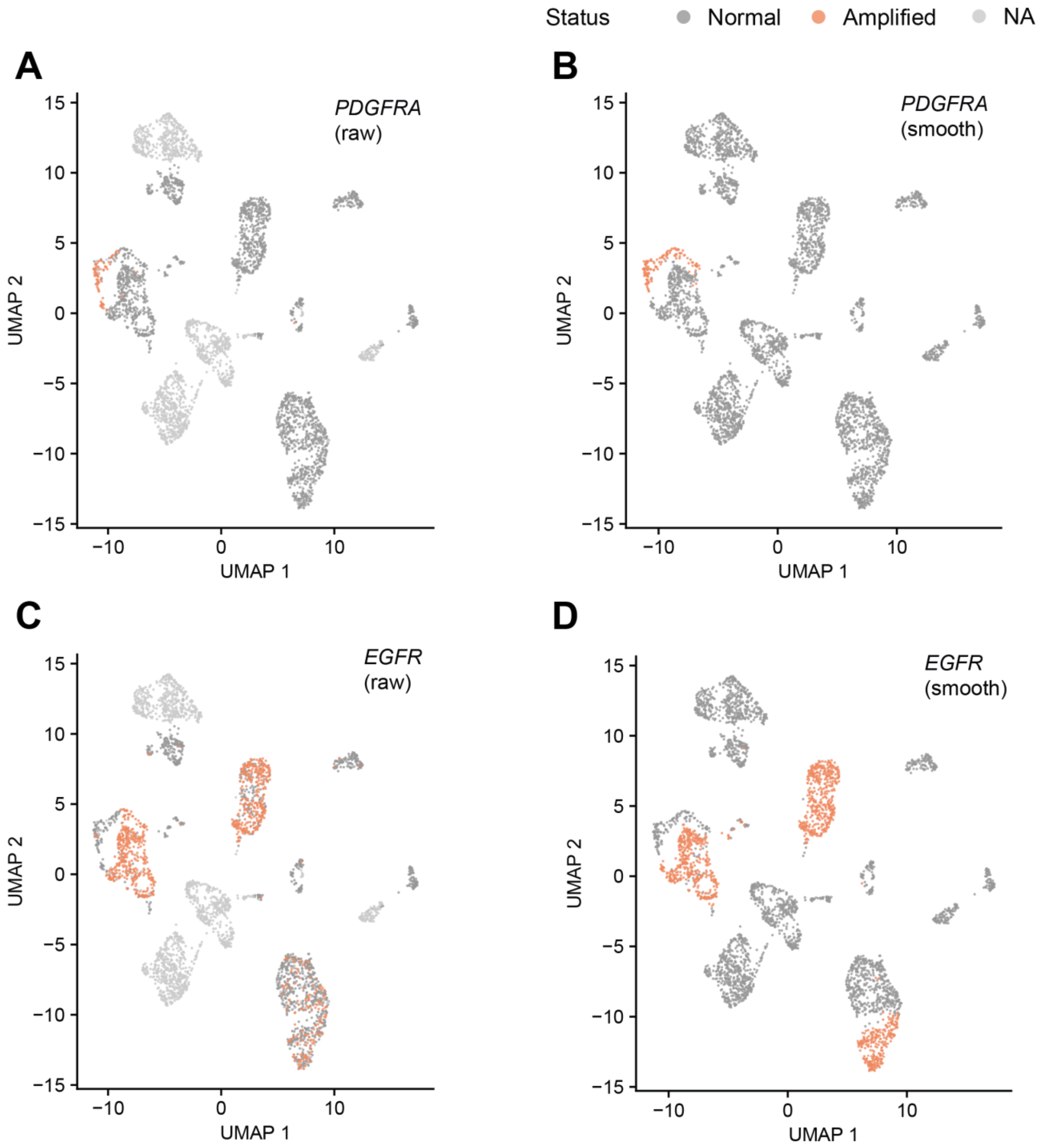

Fig. S8. CNVs are detected in scATAC clusters with Copy-scAT in pediatric GBM samples. **(A)** Overview of cell assignments in two paired patient libraries. **(B)** Overview of signal clusters used for sample smoothing of patient libraries. **(C-D)** Representative WGS-confirmed alterations detected in paired samples pCGY2932 and pCGY2937.

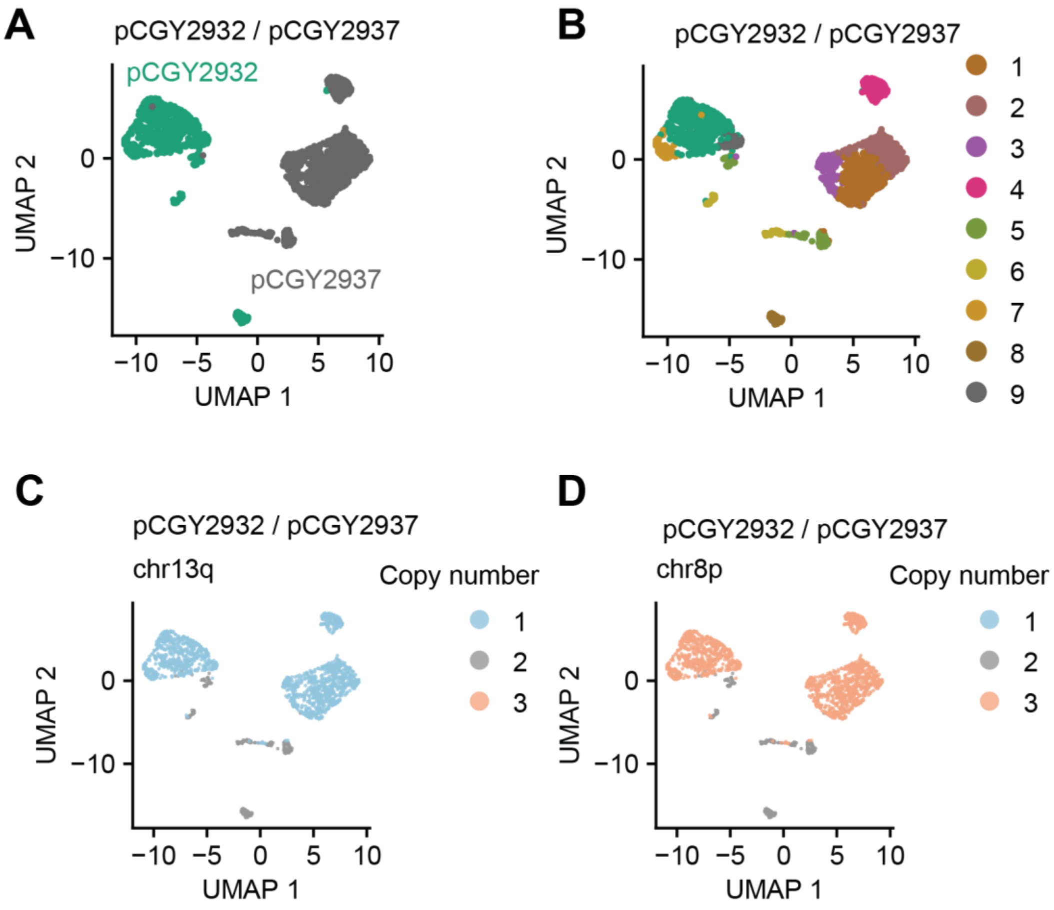

Fig. S9. Additional chromosome copy number analyses for CGY4218.
(A) Initial neighbourhood clustering results from Signac.
(B-F) Representative chromosome-level copy number alteration profiles for tumour and normal cells. **(G-N)** Representative motif scores from ChromVAR for different motifs, including **(G)** ELF5, **(H)** SPIB, **(I)**, ASCL1, **(J)** IKZF1, **(K)** NEUROD1, **(L)** NFIC, **(M)** NFYA, **(N)** ELK3.

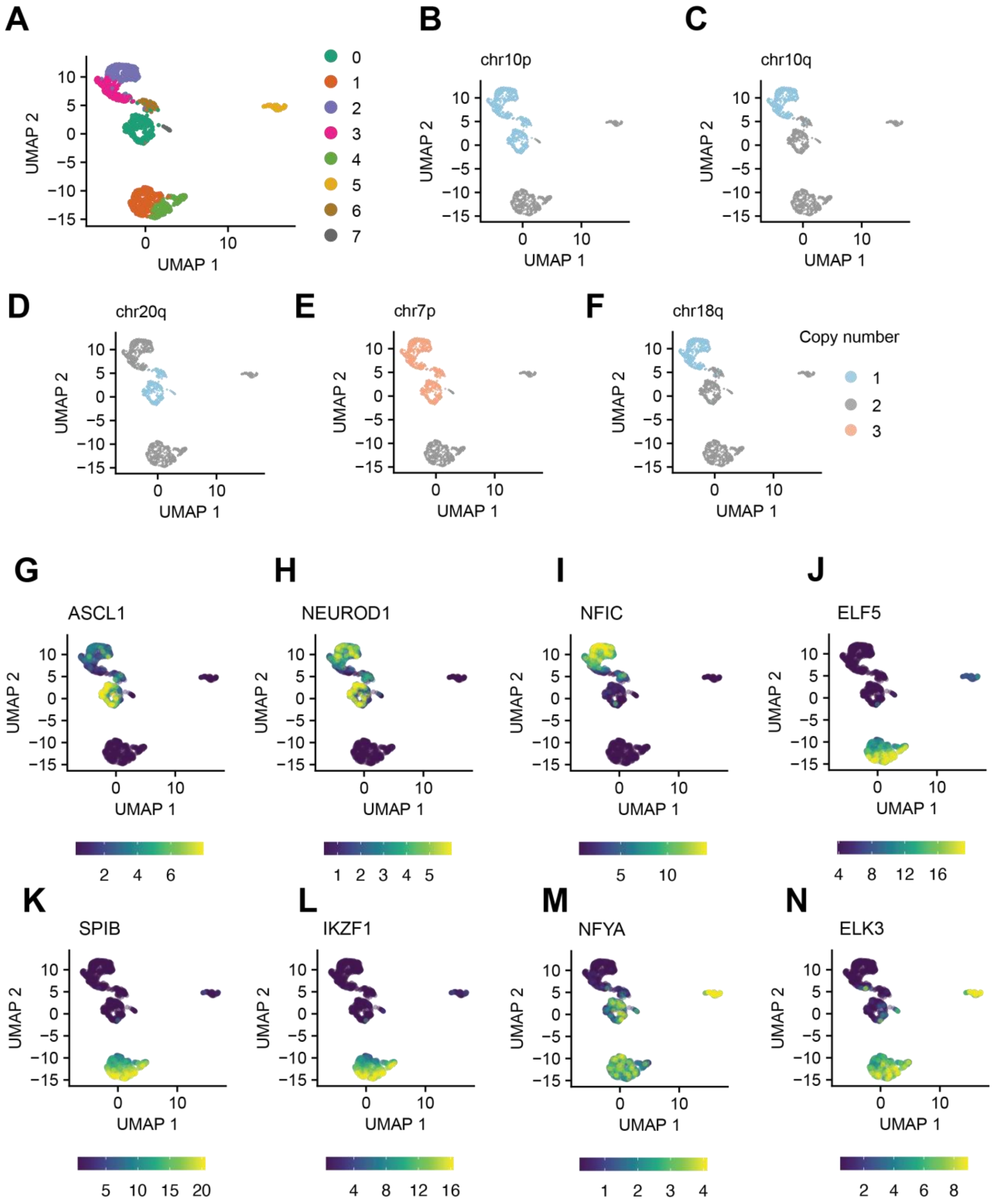

Fig S10. Representative copy number information and distribution for aGBM sample CGY4250.

(A) Neighbourhood clustering results from Signac. **(B-C)** Distribution of amplifications in *EGFR* and *MDM2*. **(D-I)** Representative chromosome-level copy number alteration profiles for tumour and normal cells. **(J-L)** Representative motif scores from ChromVAR for different motifs, including **(J)** NFIC, **(K)** SPIB and **(L)** FOXG1.

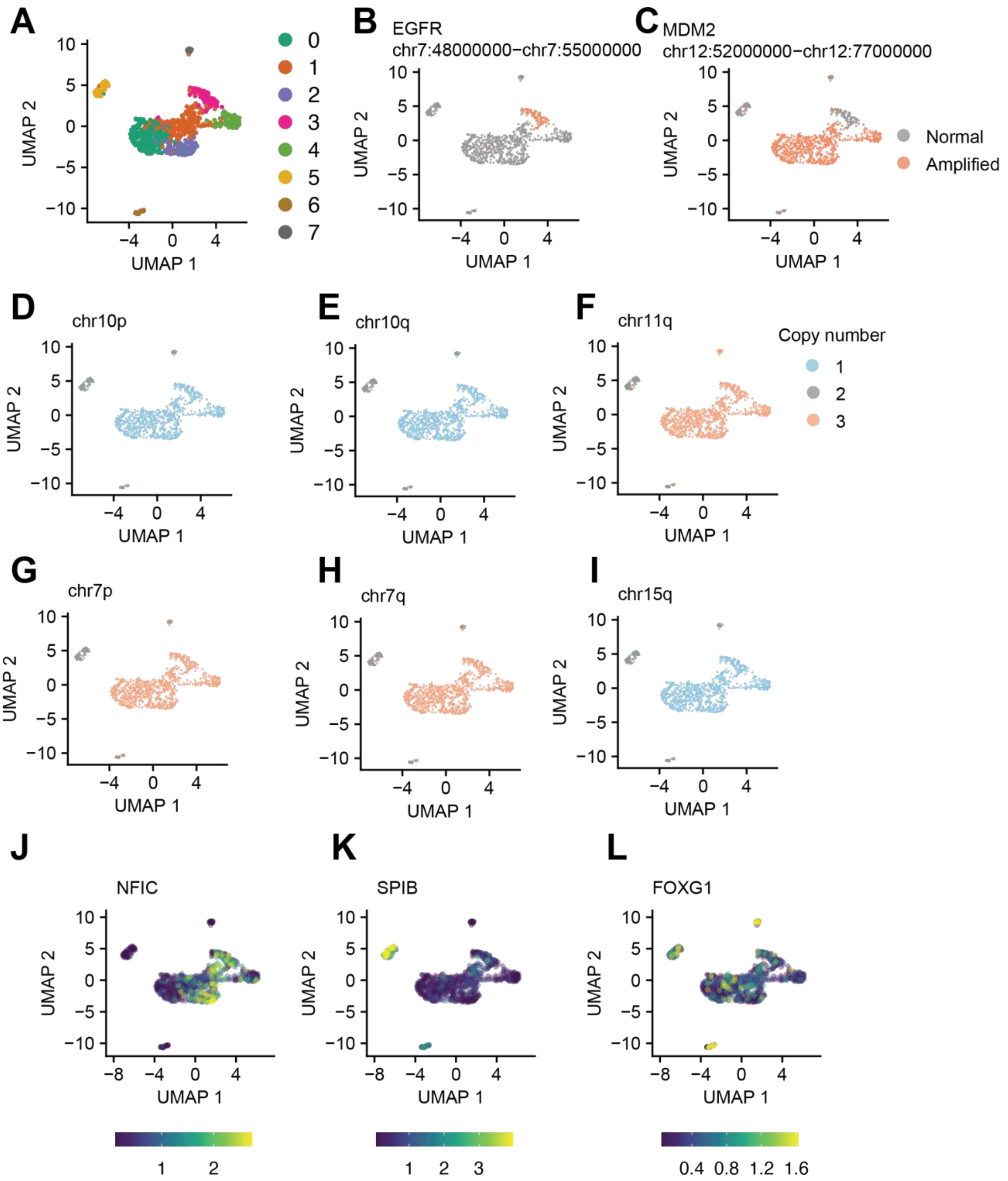
A
B
EGFR
chr7:48000000−chr7:55000000
C
MDM2
chr12:52000000−chr12:77000000
Normal
Amplified
D
chr10p
E
chr10q
F
chr11q
Copy number
1
2
3
G
chr7p
H
chr7q
I
chr15q
J
NFIC
K
SPIB
L
FOXG1
UMAP 1
UMAP 2

Fig S11. Representative copy number information and distribution for aGBM sample CGY4275. **(A)** Neighbourhood clustering results from Signac. **(B)** Distribution of amplifications in *EGFR*. **(C-F)** Representative chromosome-level copy number alteration profiles for tumour and normal cells. **(G-L)** Representative motif scores from ChromVAR for different motifs, including **(G)** NFIC, **(H)** FOS::JUN, **(I)** NEUROD1, **(J)** ELF5, **(K)** SPIB, and **(L)** IKZF1.

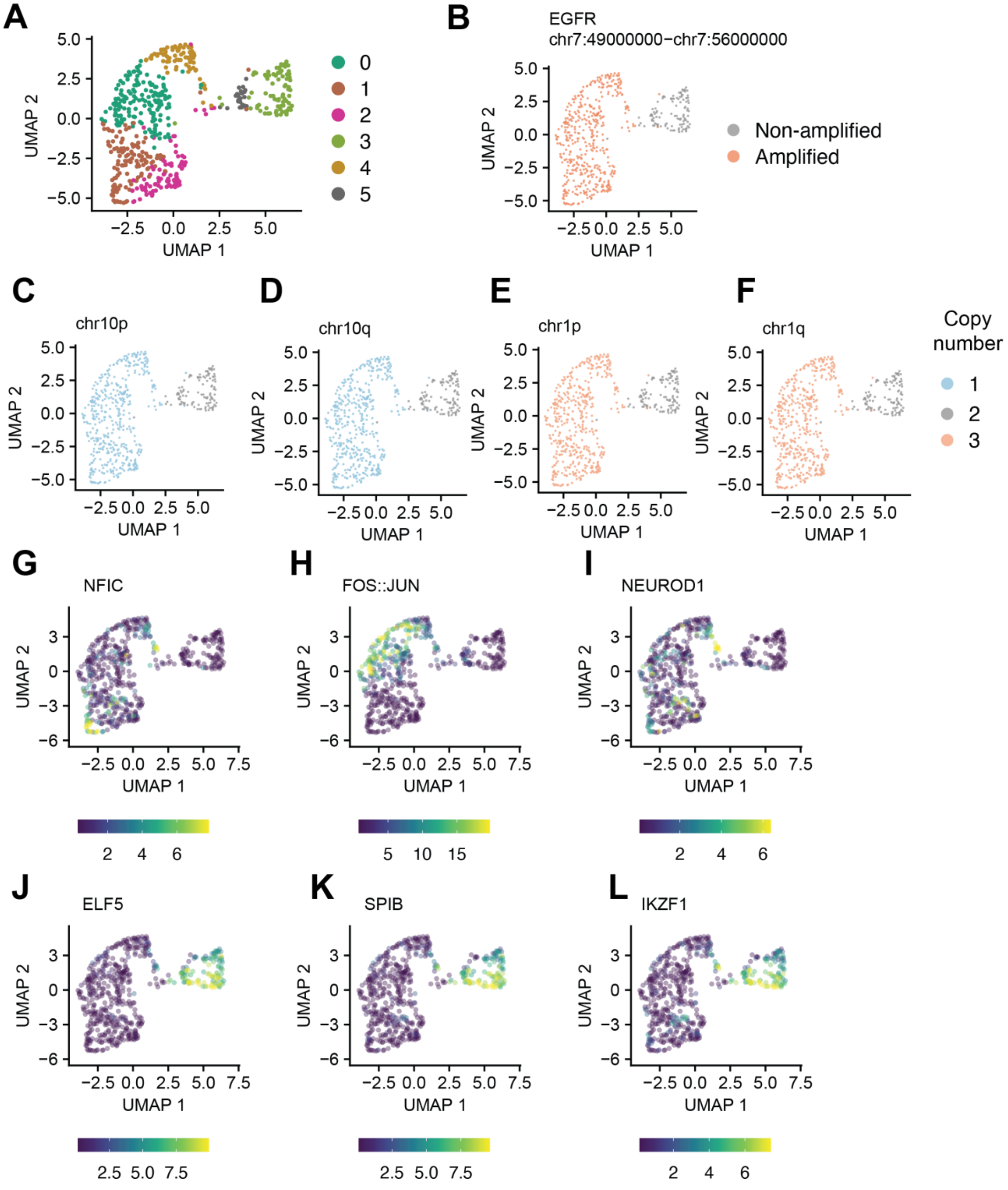

Fig S12. Comparison of identification of putative neoplastic and non-neoplastic cells with ChromVAR versus Copy-scAT. (A) Consensus clusters for adult GBM samples using ChromVAR. **(B)** Consensus status for adult GBM samples using CNVs based on Copy-scAT calls.

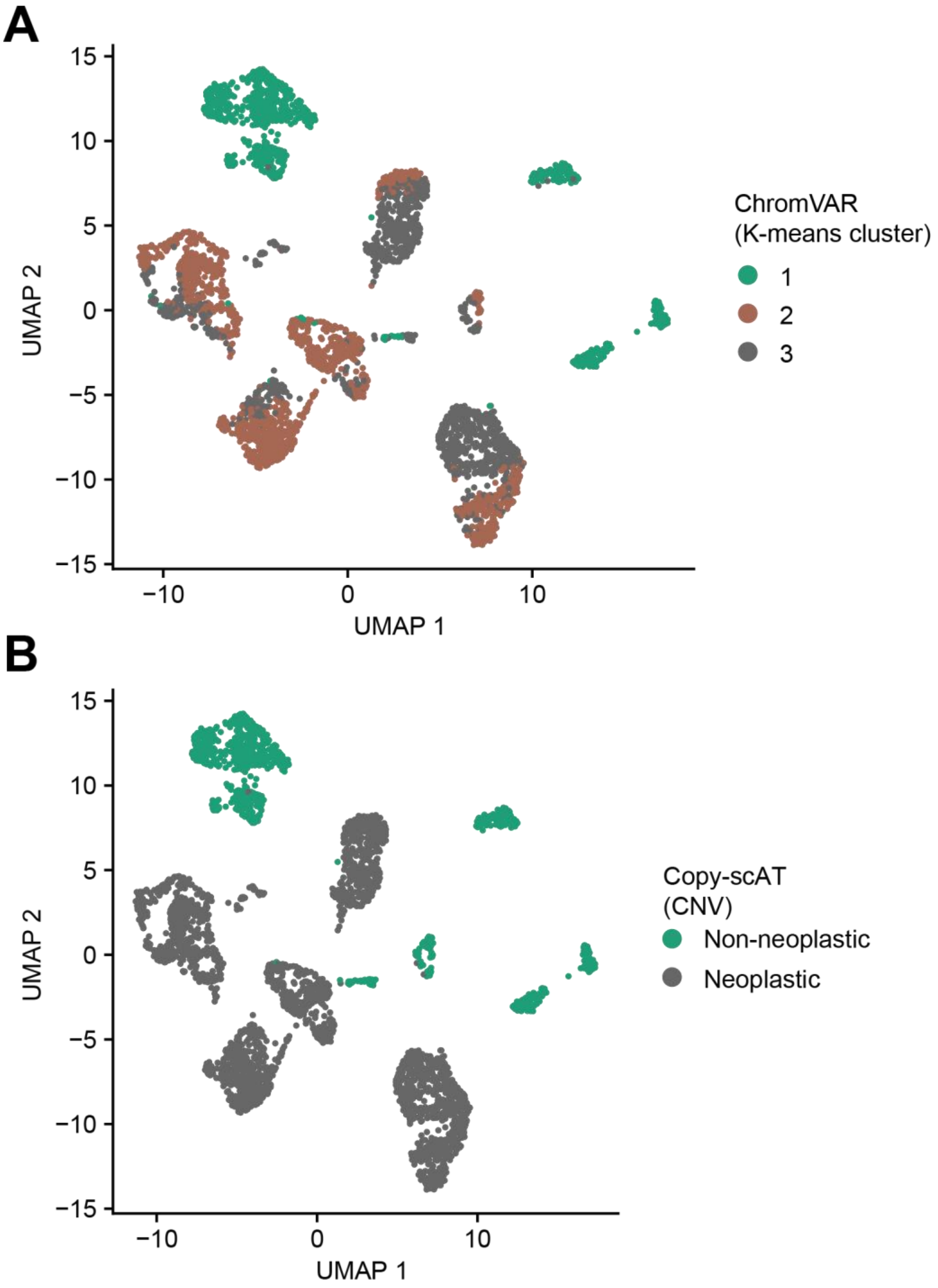

Fig. S13. Effects of removing CNVs on variance in aGBM sample CGY4349.
(A) Distribution of the top 2000 most variable peaks in the tumour cells after filtering out non-neoplastic cells; p value from Chi-squared test. **(B)** Distribution of top 2000 most variable peaks in non-neoplastic cells after filtering (p value from chi-squared test). Chromosomes with CNVs or amplification regions are highlighted in pink. **(C)** Distribution of top 2000 most variable peaks in tumour cells after filtering of non-neoplastic cells and removal of regions containing CNVs (p value from chi-squared test).

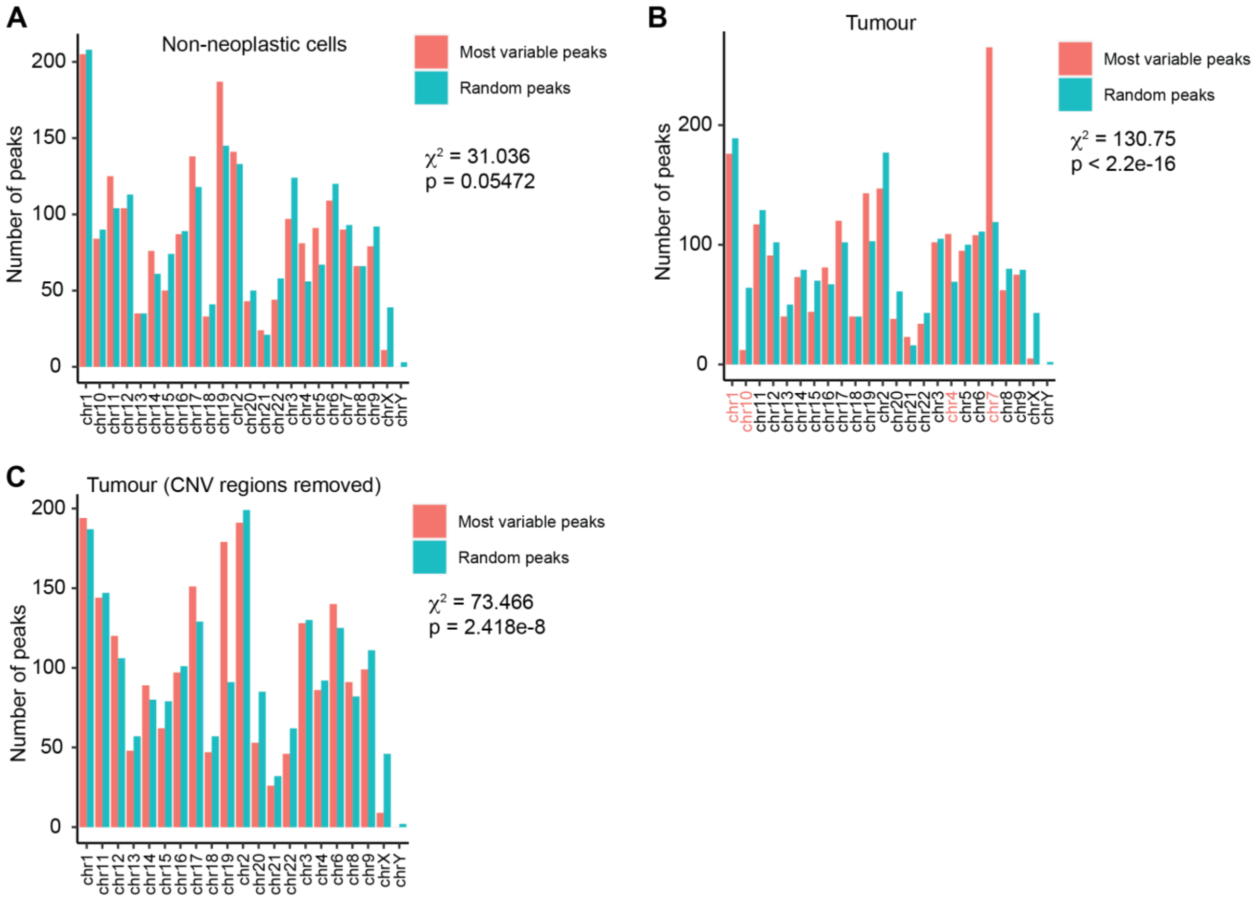

Fig. S14. Validation of Copy-scAT and identification of putative proliferative cells in non-neoplastic datasets.
(A) Chromosome copy number distribution in a 10X dataset of 5000 human PBMCs. **(B)** Seurat clusters for the 10X dataset of 5000 human PBMCs. **(C)** Estimate of cycle status for the 10X dataset of 5000 human PBMCs. **(D)** Chromosome copy number distribution in a 10X dataset of mouse embryonic brain at E18. **(E,F)** Predicted cycle status and cluster assignments in E18 mouse brain. **(G,H)** Predicted cell cycle status and cluster profile in P50 mouse brain dataset from 10X.

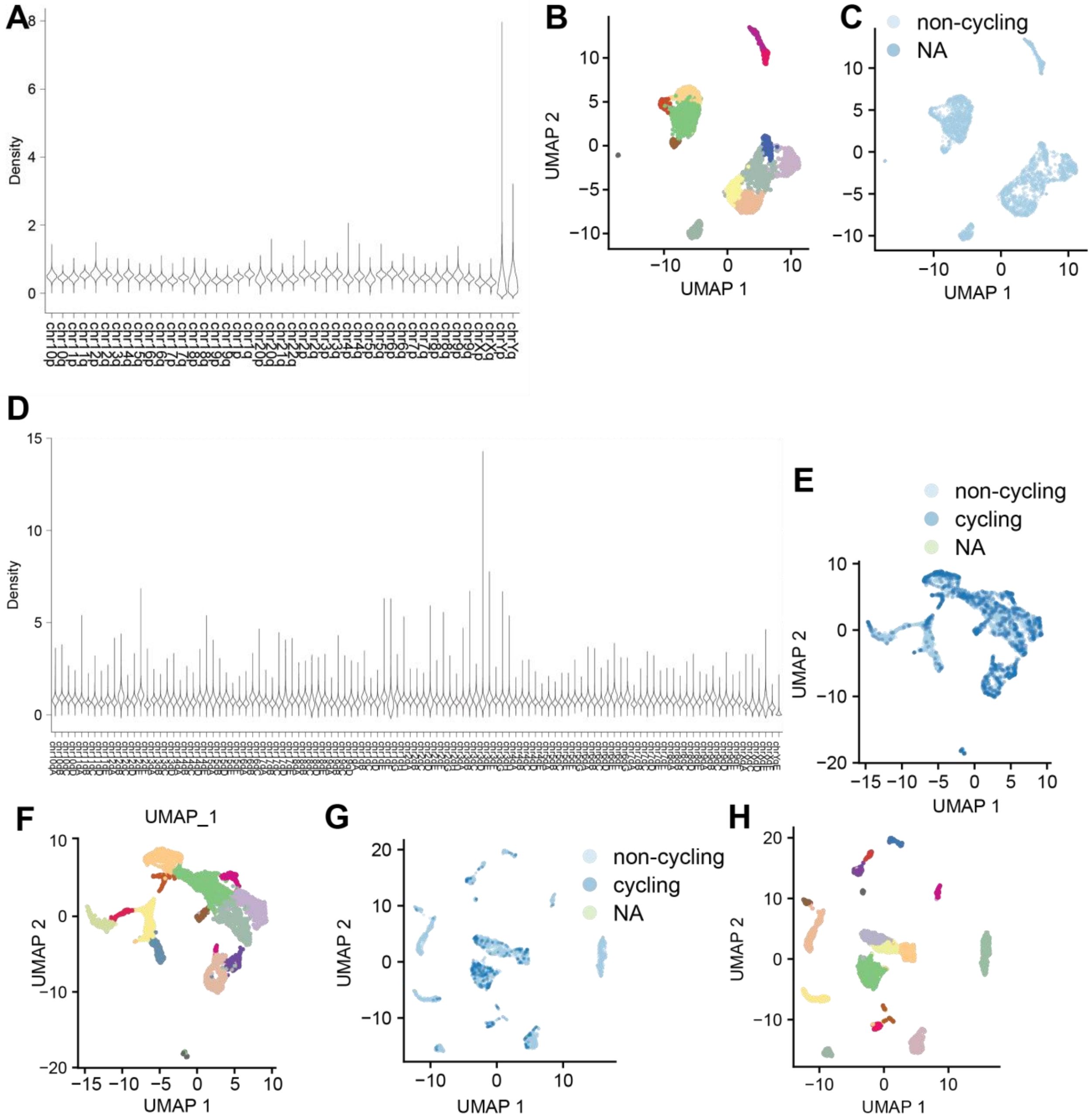

Fig S15. Cell cycle status of CGY4218 glioblastoma cells separated by branch. (A) Proportions of cycling cells in all 3 branches of CGY4218 adult glioblastoma. P-value: Chi-square test.

A

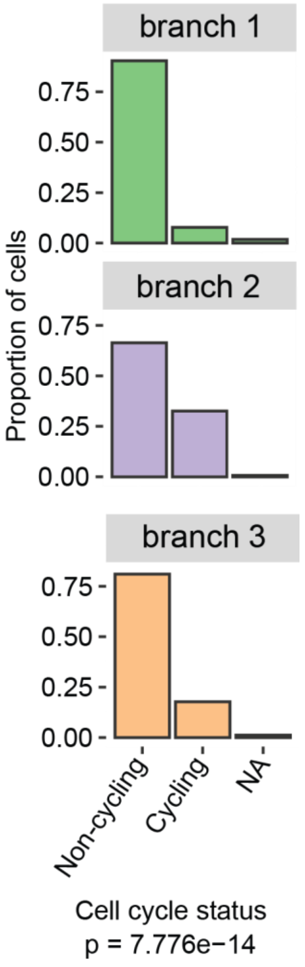

Fig. S16. Pseudotime trajectory analysis of aGBM sample CGY4250.
Distribution of EGFR amplification **(A)** and cell cycle status **(B)** amongst branches. Distribution of ChromVAR motif scores in branches for proneural motifs ASCL1 and OLIG2 **(C,D)**, ETV1 **(E)**, NFIX **(F)**, and mesenchymal motifs JUN::JUNB **(G)** and STAT3 **(H)**.

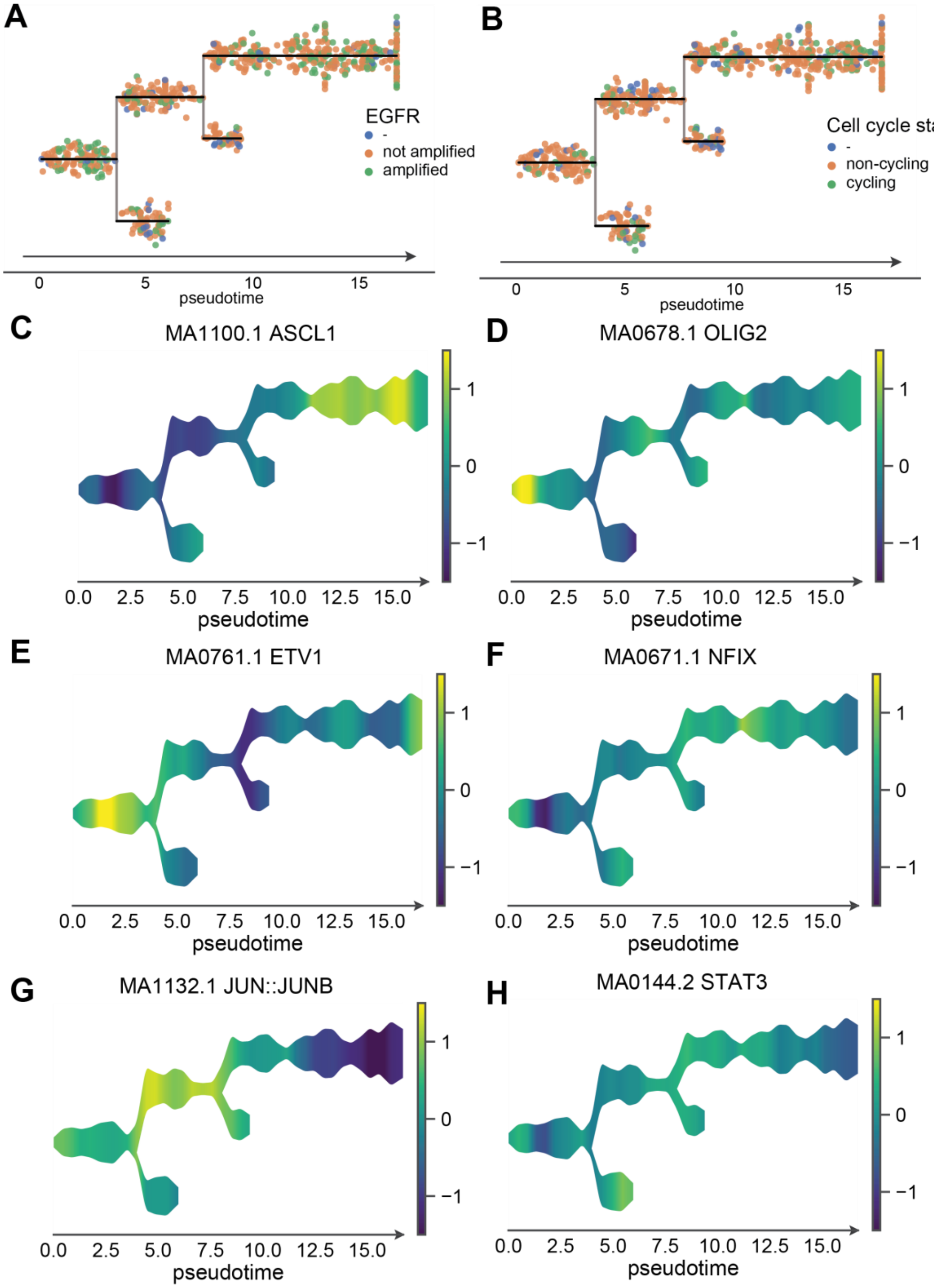

Fig. S17. Pseudotime trajectory analysis of aGBM sample CGY4349.
Distribution of PDGFRA amplification **(A)** and cycling status **(B)** amongst branches. Distribution of ChromVAR motif scores in branches for proneural motifs ASCL1 and OLIG2 **(C,D),** ETV1 **(E)**, NFIX **(F)**, and mesenchymal motifs JUN::JUNB **(G)** and STAT3 **(H)**.

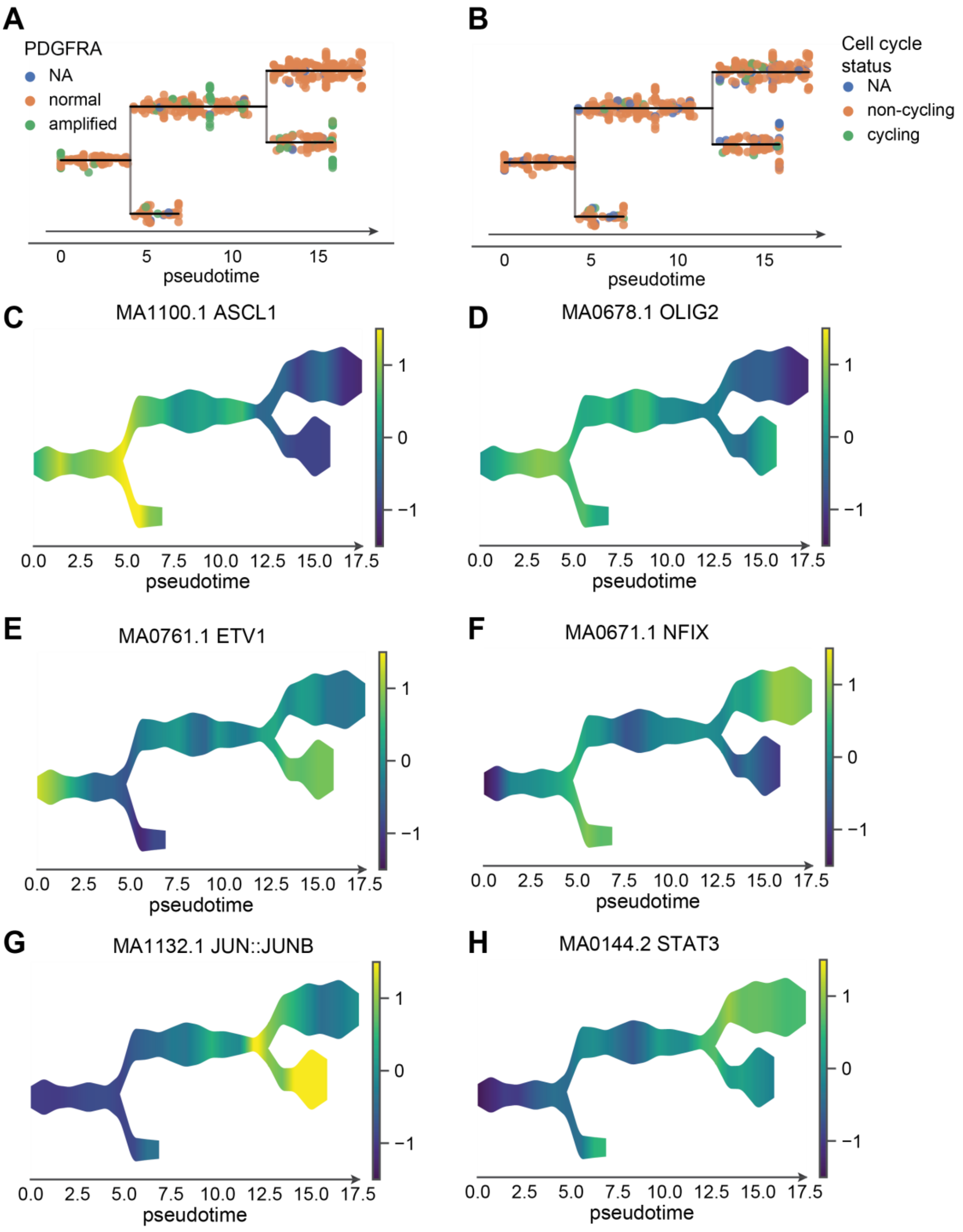

Fig S18. Pseudotime trajectory analysis of aGBM sample CGY4275.
Distribution of ChromVAR motif scores in branches for proneural motifs ASCL1 and OLIG2 **(A,B)**, ETV1 **(C)**, NFIX **(D)**, and mesenchymal motifs JUN::JUNB **(E)** and STAT3 **(F)**.

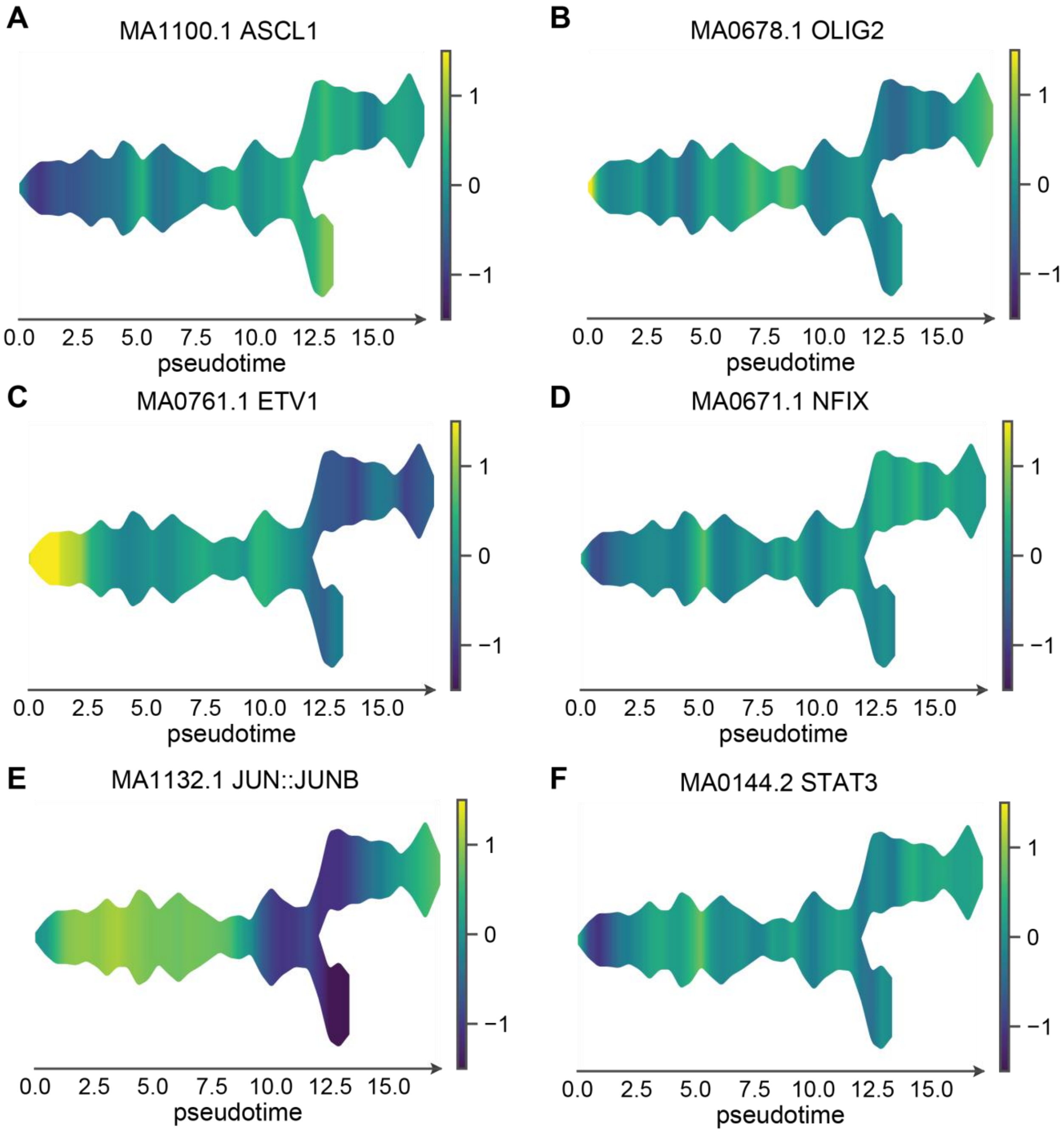

Supplement: Supplementary file 1 — Tables S1 and S2 Figs. S1 to S18 [file sciadv.abg6045_sm.pdf]
